# Supplementary material for: A small-molecule inhibitor of the BRCA2-RAD51 interaction modulates RAD51 assembly and potentiates DNA damage-induced cell death
Source: Cell Chem Biol. 2021 Jun 17;28(6):835–847.e5. doi: 10.1016/j.chembiol.2021.02.006 (PMC8219027; doi:10.1016/j.chembiol.2021.02.006)
Supplement: Document S1. Figures S1–S5, Tables S1–S3, and Methods S1 [file mmc1.pdf]

Cell Chemical Biology, Volume *28*

Supplemental information

A small-molecule inhibitor of the BRCA2-RAD51 interaction modulates RAD51 assembly and potentiates DNA damage-induced cell death

Duncan E. Scott, Nicola J. Francis-Newton, May E. Marsh, Anthony G. Coyne, Gerhard Fischer, Tommaso Moschetti, Andrew R. Bayly, Timothy D. Sharpe, Kalina T. Haas, Lorraine Barber, Chiara R. Valenzano, Rajavel Srinivasan, David J. Huggins, Miyoung Lee, Amy Emery, Bryn Hardwick, Matthias Ehebauer, Claudio Dagostin, Alessandro Esposito, Luca Pellegrini, Trevor Perrior, Grahame McKenzie, Tom L. Blundell, Marko Hyvönen, John Skidmore, Ashok R. Venkitaraman, and Chris Abell

Supplementary material

A small-molecule inhibitor of the BRCA2-RAD51 interaction modulates RAD51 assembly and potentiates DNA damage-induced cell death.

Duncan E. Scott, Nicola J. Francis-Newton, May E. Marsh, Anthony G. Coyne, Gerhard Fischer, Tommaso Moschetti, Andrew R. Bayly, Timothy D. Sharpe, Kalina T. Haas, Lorraine Barber, Chiara R. Valenzano, Rajavel Srinivasan, David J. Huggins, Miyoung Lee, Amy Emery, Bryn Hardwick, Matthias Ehebauer, Claudio Dagostin, Alessandro Esposito, Luca Pellegrini, Trevor Perrior, Grahame McKenzie, Tom L. Blundell, Marko Hyvönen, John Skidmore, Ashok R. Venkitaraman, Chris Abell.

Table of Contents

Supplementary material 1

Figure S1. ITC data for the binding of **3** (left, K_d 1.3 mM) and **4** (right, K_d 3 µM) to HumRadA2 (related to figure 2)........ 2

Figure S2. ITC data for the binding of **5** to HumRadA2 (K_d 220 nM) (related to figure 2). 2

Figure S3. Structure of **6** bound to two forms of humanised RadA (related to figure 2). 3

Figure S4. Inhibition of RAD51 oligomerisation by CAM833 (related to figure 2). 3

Figure S5. Structure of **CAM833** bound to HumRadA22F (related to figure 2). 4

Table S1. Calculated and measured ADMET and developability properties for **CAM833** (related to figure 2 and STAR methods)........ 5

Table S2. CAM833 growth inhibition data for a range of cancer-derived human cell lines (related to figure 5)........ 6

Table S3 Crystallographic data collection and refinement statistics (related to figures 2, S3 and S5). 7

Methods S1 – chemical synthesis (related to figure 2). 8

Solvents and Reagents 8

Chromatography........ 8

Nuclear Magnetic Resonance Spectroscopy 8

Abbreviations 8

LCMS 8

Synthetic methods 8

General procedure A........ 8

General procedure B 9

(*S*)-1-((2-Naphthoyl)-L-alanyl)-*N*-((*S*)-1-phenylethyl)pyrrolidine-2-carboxamide (5) 9

N-(2-((*S*)-2-(((*S*)-1-(4-methoxyphenyl)ethyl)carbamoyl)pyrrolidin-1-yl)-2-oxoethyl)quinoline-2-carboxamide (6)........ 10

N-(2-((2*S*,4*R*)-2-(((*S*)-1-(2-chloro-4-methoxyphenyl)ethyl)carbamoyl)-4-hydroxypyrrolidin-1-yl)-2-oxoethyl)-6-fluoroquinoline-2-carboxamide CAM833A 11

Figure S1. ITC data for the binding of **3** (left, K_d 1.3 mM) and **4** (right, K_d 3 µM) to HumRadA2 (related to figure 2)

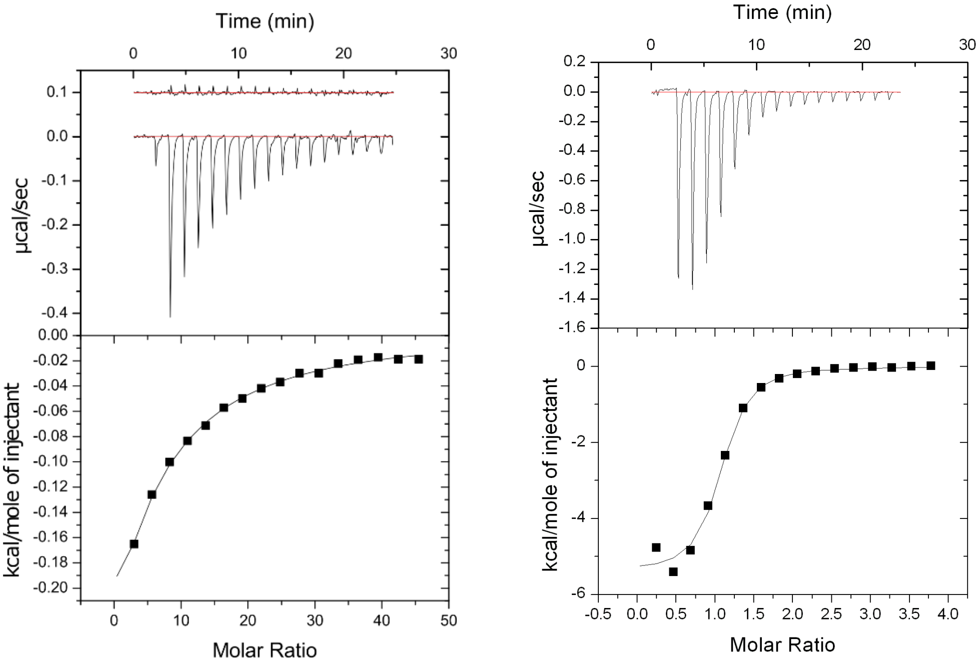

Figure S2. ITC data for the binding of **5** to HumRadA2 (K_d 220 nM) (related to figure 2).

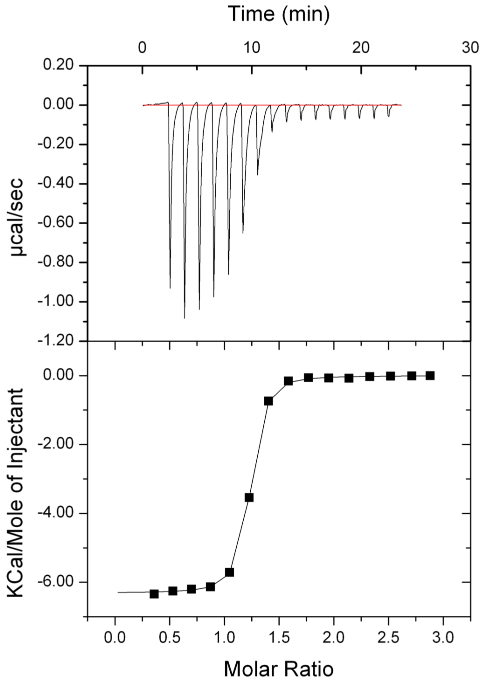

Figure S3. Structure of **6** bound to two forms of humanised RadA (related to figure 2).

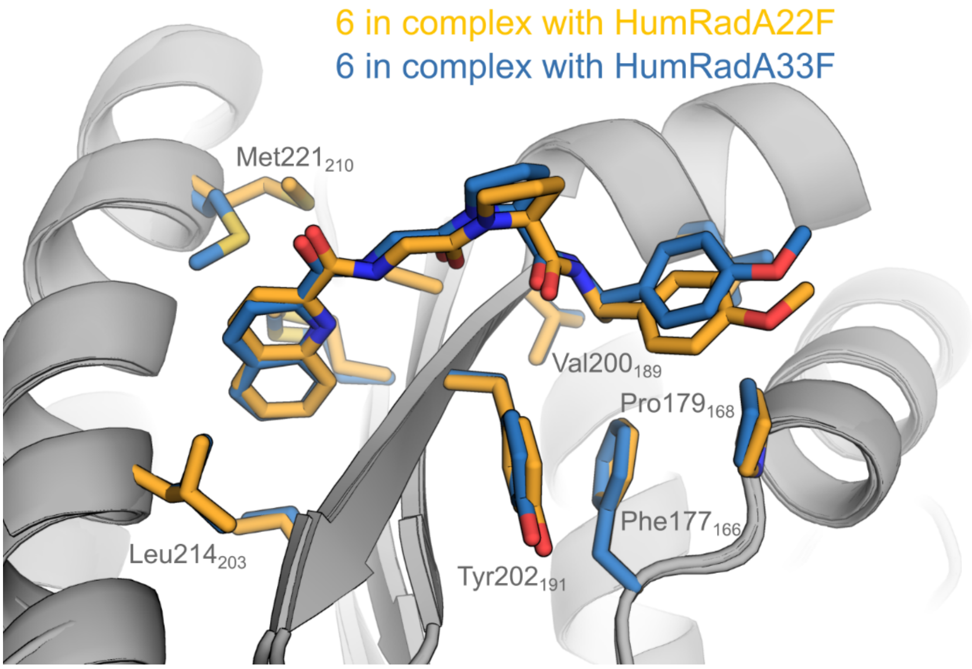

HumRadA22F (orange carbons, PDB:6TW4) and HumRadA33F (blue carbons, PDB: 6XTW) in complex with **6** with the side chains of residues around the Phe and Ala pockets shown as sticks and in the same colour as the ligand bound to that protein.

Figure S4. Inhibition of RAD51 oligomerisation by CAM833 (related to figure 2).

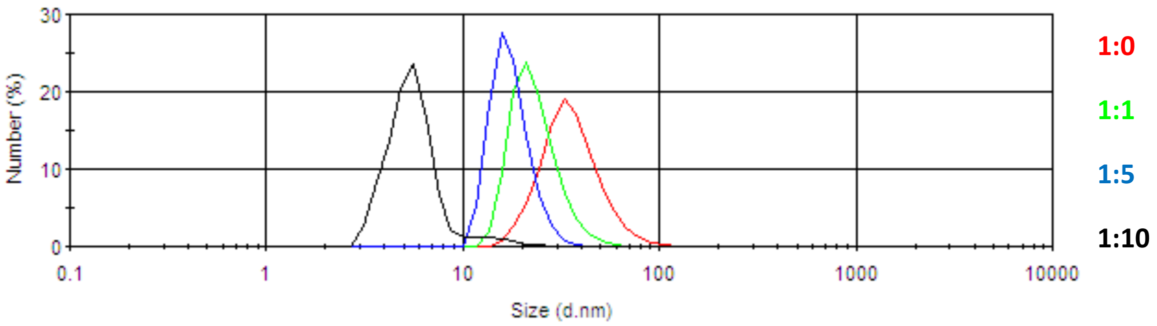

Particle size distribution (in nm) of full-length RAD51 protein in the absence (red line) and presence of increasing concentrations of CAM833. Green line indicates 1:1 stoichiometry of RAD51:CAM833, blue line is for sample with 1:5 stoichiometry and the black line for 1:10 stoichiometry of the components.

Figure S5. Structure of **CAM833** bound to HumRadA22F (related to figure 2).

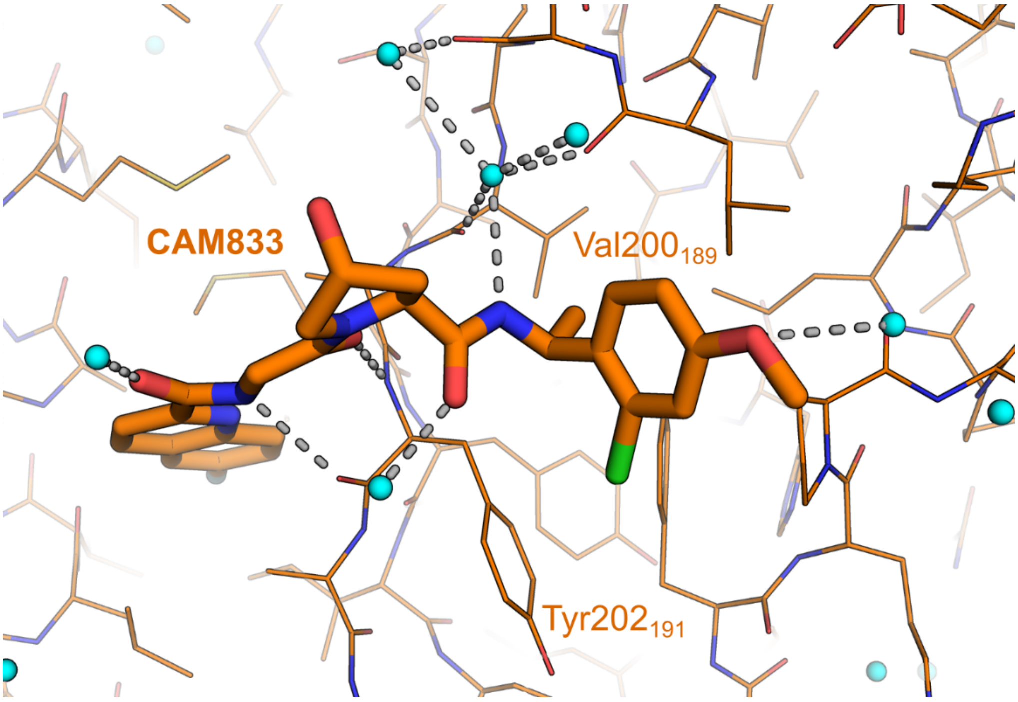

CAM833, in thicker sticks, is bound to HumRadA22F with hydrogen bonds between the inhibitor and the protein (thinner lines) or waters (small cyan spheres) shown as grey dotted lines.

Table S1. Calculated and measured ADMET and developability properties for CAM833 (related to figure 2 and STAR methods).

MW	529
PSA	120
clogP[a]	2.73
K_D FP (ChimRAD51)	355 nM,[b] (n=8)
Aqueous solubility [c]	7.75 µg/ml (n=2, s.d. 0.549)
Microsomal CL_{int} [d]	Mouse $t_{½}$ 43.1 min<br>32.2 ± 2.99 µL/min/mg protein<br>Rat $t_{½}$ 47.9 min<br>28.9 ± 4.16 µL/min/mg protein<br>Human $t_{½}$ 76.8 min<br>18 ± 4.52 µL/min/mg protein
CYP450 IC_{50} (1A2, 2C9, 2C19, 2D6, 3A4 midazolam and testosterone sites)	all > 25 µM
hERG electrophysiology @ 10 uM[e]	12%
Human plasma protein binding [f]	99.2% bound
Caco-2 permeability<br>AtoB P_{app} (10^{-6} cm s^{-1}) / Efflux Ratio	0.23 / 195
Selectivity Cerep ExpresSPanel	no inhibition of binding >50% at 10 µM
Mouse iv pharmacokinetics at 1 mg/kg[g]	t½ = 1.44 h (n=2)<br>CL 44.6 ml/min/kg (n=3)<br>Vd_{ss} 2.51 L/kg (n=2)
Mouse oral screen at 50 mg/kg [h]	Cmax 8323 ng/ml (n=3)<br>F = 44%

a. Calculated using Chemdraw 16

b. pK_d = 6.45 ± 0.16, n=8 determined by FP

c. Thermodynamic solubility from solid, determined overnight in pH 7.4 buffer.

d. Intrinsic clearance calculated from 5 timepoints over a 45 minute experiment reported as half-life and intrinsic clearance ± standard error

e. Inhibition of hERG tail-currents measured by whole-cell voltage-clamping in mammalian cells.

f. Determined by equilibrium dialysis

g. Fasted male CD-1 mice. 0.5 mg/mL in 20%HP-β-CD in water, clear solution. n=3 or 2

h. Fasted male CD-1 mice, fasted. 5 mg/mL in 70% PEG400 / 30% water, clear solution. n=3 or 2.

Table S2. CAM833 growth inhibition data for a range of cancer-derived human cell lines (related to figure 5).

Cell Line	Absolute EC_{50} (µM)
HUVEC	133.0
Mia-pa-ca2	68.6
NCI-H209	69.3
OVCAR3	53.6
PC3	91.2
SK-MEL-24	143.5
U20S	40.5
BT549	73.8
HCT116	63.5
A375	72.0
BT20	61.9
HT1367	104.0
MDA-MB-453	48.0
RT112/84	70.6
T.Tn	55.8
U118MG	90.3
SCaBER	47.0
SK-OV-3	104.8
T24/83	96.7
U87	108.8
A549	39.0
Calu3	83.3
HepG2	68.1
HFL-1	98.8
HL-60	87.7
K562	137.1
LN229	117.3
Raji	95.6
RKO	63.5
5637.00	84.5
A2780	46.5
Caki1	73.0
PANC1	96.0
SW756	124.3

Table S3 Crystallographic data collection and refinement statistics (related to figures 2, S3 and S5).

Ligand	**3**	**4**	**6**	**6**	**CAM833**
Protein form	HumRadA1	HumRadA1	HumRadA22F	HumRadA33F	HumRadA22F
PDB code:	6TV3	6TWR	6TW4	6XTW	6TW9
**Data Collection and Processing:**					
Synchrotron beamline	ESRF ID14-4	DLS I04	DLS I24	DLS I03	DLS I02
Wavelength (Å)<br>Resolution range (Å)<br>(High resolution bin)	0.9795<br>21.03 - 1.50<br>(1.59 - 1.50)	0.9702<br>31.63 - 1.35<br>(1.43 - 1.35)	0.9686<br>40.39 - 1.73<br>(1.77 - 1.73)	0.9300<br>100.82-2.31<br>(2.43-2.31)	0.9795<br>61.21 - 1.52<br>(1.522 - 1.517)
Space group	P 1 21 1	P 21 21 21	P 21 21 21	P31 2 1	P 21 21 21
Unit cell (a b c) (Å)	37.67 79.24 39.43	40.53 61.95 87.72	40.39 60.15 88.20	89.83 89.83 100.82	40.38 61.21 87.68
Unit cell (α β γ) (°)	90.00 118.18 90.00	90.00 90.00 90.00	90.00 90.00 90.00	90.00 90.00 120.00	90.00 90.00 90.00
Total number of reflections	104241 (31919)	325400 (44541)	182953 (12814)	107445 (16048)	149322 (1612)
Number of unique reflections	31919 (5063)	48841 (7575)	23054 (1639)	21180 (3039)	32571 (351)
Multiplicity	3.7 (3.6)	6.7 (5.9)	7.9 (7.8)	5.1 (5.3)	4.6 (4.6)
Completeness (%)	97.4 (96.4)	99.5 (96.9)	99.6 (98.6)	99.9 (99.9)	94.6 (97.5)
Mean I/sigma(I)	15.9 (2.9)	13.8 (2.2)	15.2 (0.6)	14.4 (2.8)	12.5 (2.2)
R_{merge}	0.049 (0.45)	0.080 (0.64)	0.087 (1.25)	0.067 (0.621)	0.087 (0.67)
R_{pim}	- (-)	- (-)	0.035 (0.50)	0.036 (0.330)	0.043 (0.34)
CC-half	0.999 (0.87)	0.998 (0.77)	- (-)	- (-)	- (-)
**Refinement:**					
R / R_{free}	0.183 / 0.217	0.187 / 0.210	0.178 / 0.210	0.184 (0.229)	0.156 / 0.172
Number of atoms	1985	2334	1955	3664	3853
No. of ligand atoms	40	75	35	35	44
No. of waters	221	358	143	118	258
No. of protein residues	219	227	226	227	224
Average/Wilson<br>B-factor (Å^2)	24.5 / 18.5	18.2 / 19.3	27.8 / 23.2	48.0	17.0 / 15.2
B-factor for ligands (Å^2)	34.8	26.4	28.6	52.0	19.1
B-factor for solvent (Å^2)	39.3	29	39	46.9	30.6
RMS (bonds) (Å)	0.01	0.005	0.01	0.007	0.011
RMS (bond angles)	1.12	0.81	1.09	0.862	1.16

Methods S1 – chemical synthesis (related to figure 2).

Solvents and Reagents

Unless otherwise stated starting materials and reagents were purchased from regular suppliers. Dry solvents were purchased and used as provided.

Chromatography

Thin layer chromatography (TLC) was performed on glass plates coated with Merck 60 F254 silica and visualization was achieved by UV light or by staining potassium permanganate. Flash column chromatography was using a Biotage Isolera One and Biotage Isolera Four systems with UV detection at 254 nm and 280 nm and commercially available cartridges.

Nuclear Magnetic Resonance Spectroscopy

^{1}H NMR spectra were recorded on a Bruker Avance 400 (400 MHz), or Bruker Avance Cryo 500 (500 MHz). Chemical shifts are quoted in ppm and are referenced to the residual non-deuterated solvent peak, and are reported (based on appearance rather than interpretation) as follows: chemical shift δ /ppm (multiplicity, coupling constant J/Hz, number of protons) [br, broad; s, singlet; d, doublet; t, triplet; q, quartet; qui, quintet; sept, septet; m, multiplet]. All J values are given in Hz. Fractional integrations are reported where conformational restriction of peptidic compounds results in separate signals on the nmr timescale.

Abbreviations

DCM - dichloromethane
DMAP – 4-dimethylaminopyridine
EDAC - *N*-(3-dimethylaminopropyl)-*N′*-ethylcarbodiimide hydrochloride
HBTU - *N,N,N′,N′*-tetramethyl-*O*-(1*H*-benzotriazol-1-yl)uronium hexafluorophosphate
PyBOP - benzotriazole-1-yl-oxy-tris-pyrrolidino-phosphonium hexafluorophosphate
THF – tetrahydrofuran

LCMS

High-resolution mass measurements were performed on a Waters LCT Premier mass spectrometer or a Kratos Concept mass spectrometer. Low-resolution measurements were recorded on a Waters / ZQ LCMS and on a Waters Acquity UPLC HClass LCMS. All final compounds used for screening and cell experiments were at least 95% pure as determined by LCMS unless otherwise stated.

Synthetic methods

Tetrapeptide (**1**) was prepared as described previously (Scott et al., 2016). Fragments 2-naphthol (**2**) and 3-amino-2-naphthoic acid (**3**) are commercially available and used as supplied. Tetrapeptide **(4)** was prepared using standard solid phase chemistry with Fmoc protection by the Protein and Nucleic Acid Service at the Department of Biochemistry (University of Cambridge).

General procedure A

EDAC (1.5 equiv.) was added to a stirred solution of acid (1 equiv.), amine (2 equiv.), *N*-methylmorpholine (3 equiv.) and DMAP (1 equiv.) in DCM (0.05 M) and stirred until the reaction was judged complete. The solvent was removed *in vacuo* and DCM and H_2O were added to the residue, the organic layer was separated, washed with H_2O, brine, dried with $MgSO_4$, filtered and the solvent removed *in vacuo*. The residue was purified by flash chromatography (FC) (2-20% MeOH: DCM) to give the product.

General procedure B

To a solution of acid (1 equiv.), amine (1-1.1 equiv.) and DIPEA (2-5 equiv.) in solvent was added PyBOP (1.1-1.4 equiv.) and the reaction was stirred until complete and then concentrated *in vacuo*. Unless stated otherwise, the crude was diluted with ethyl acetate and the organics were washed three times with water, and then brine and dried before purifying typically by FC as described to give the product.

(*S*)-1-((2-Naphthoyl)-L-alanyl)-*N*-((*S*)-1-phenylethyl)pyrrolidine-2-carboxamide (5)

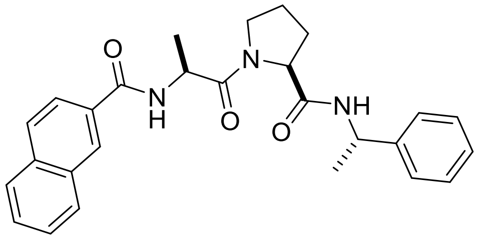

Methyl (2-naphthoyl)-L-alanyl-L-prolinate
A mixture of methyl L-alanyl-L-prolinate hydrochloride (200 mg, 0.84 mmol), 2-naphthoic acid (218 mg, 1.27 mmol), EDAC (243 mg, 1.27 mmol), *N*-methylmorpholine (204 µL, 1.86 mmol) and DMAP (103 mg, 0.84 mmol) in DCM (10 mL) was stirred until judged complete and purified by FC (1-10% MeOH: DCM) to give the product as a white foam (240 mg, 81%).
^{1}H NMR (500 MHz, Methanol-d_4) δ 8.43 (d, *J* = 1.6 Hz, 1H), 7.98 (dd, *J* = 7.8, 1.6 Hz, 1H), 7.96 – 7.88 (m, 3H), 7.62 – 7.54 (m, 2H), 4.52 (dd, *J* = 8.6, 4.7 Hz, 1H), 3.94 (dt, *J* = 9.9, 7.1 Hz, 1H), 3.75 (dt, *J* = 10.1, 6.6 Hz, 1H), 3.72 (s, 3H), 2.36 – 2.24 (m, 1H), 2.09 (p, *J* = 6.7 Hz, 2H), 1.99 (dtd, *J* = 12.5, 6.7, 4.8 Hz, 1H), 1.51 (d, *J* = 7.1 Hz, 3H). Proline α-CH is under the solvent peak (4.83-4.88 ppm)
LCMS m/z 355.3 $(M+H)^+$

(2-Naphthoyl)-L-alanyl-L-proline
To a solution of methyl (2-naphthoyl)-L-alanyl-L-prolinate (230 mg, 0.65 mmol) in THF:H_2O (1:1, 20 mL) was added dropwise at 0 °C a solution of NaOH (104 mg, 2.6 mmol) in H_2O (1 mL). The reaction mixture was stirred for 10 mins at 0 °C, allowed to warm to rt and stirred for 2 hours. The mixture was concentrated *in vacuo*, acidified on ice to pH 2 and the resulting white suspension was extracted with DCM (4 x 100 mL). The organic extracts were combined, dried with $MgSO_4$, filtered and the solvent removed *in vacuo* to give the acid as a white foam (212 mg, 96%) which was used without further purification.
^{1}H NMR (400 MHz, Chloroform-*d*) δ 8.30 – 8.24 (m, 0.85H), 8.23 (d, *J* = 1.3 Hz, 0.15H), 7.88 – 7.72 (m, 4H), 7.61 (d, *J* = 7.6 Hz, 0.15H), 7.53 – 7.43 (m, 2H), 7.38 (d, *J* = 7.6 Hz, 0.85H), 4.98 (p, *J* = 7.0 Hz, 0.85H), 4.85 (p, *J* = 6.8 Hz, 0.15H), 4.56 (dd, *J* = 7.0, 5.7 Hz, 0.85H), 4.49 (dd, *J* = 7.5, 3.2 Hz, 0.15H), 3.84 – 3.46 (m, 3H), 2.24 (td, *J* = 7.1, 3.1 Hz, 0.15H), 2.17 – 2.11 (m, 1H), 2.07 – 1.93 (m, 1.7H), 1.90 – 1.81 (m, 0.15H), 1.81 – 1.75 (m, 1H), 1.46 (d, *J* = 6.9 Hz, 2.55H), 1.42 (d, *J* = 6.7 Hz, 0.45H).
LCMS m/z 341.2 $(M+H)^+$

(*S*)-1-((2-Naphthoyl)-L-alanyl)-*N*-((*S*)-1-phenylethyl)pyrrolidine-2-carboxamide (5)
(2-Naphthoyl)-L-alanyl-L-proline (25 mg, 0.07 mmol) was dissolved in DCM (2 mL) and cooled to 4 ˚C under N_2 gas. To the stirred solution was added EDAC (21 mg, 0.11 mmol), (S)-α-methylbenzylamine (19 µL, 0.15 mmol) and DMAP (10 mg, 0.07 mmol). The reaction was stirred for 3 hours, and the solvent was removed *in vacuo.* The residue was dissolved in ethyl acetate (100 ml), washed with water

(2 x 100 ml), washed with brine and then dried ($MgSO_4$) and the solvent was removed *in vacuo*. The crude product was purified by FC (1-10% MeOH: DCM) to give the product as a clear oil (18 mg, 54%).
^{1}H NMR (400 MHz, Chloroform-*d*) δ 8.27 (s, 1H), 7.90 – 7.82 (m, 1H), 7.82 – 7.77 (m, 3H), 7.48 (ddt, *J* = 8.0, 6.9, 5.3 Hz, 2H), 7.33 – 7.13 (m, 6H), 7.04 (d, *J* = 8.1 Hz, 1H), 5.06 – 4.87 (m, 2H), 4.54 (dd, *J* = 8.2, 2.8 Hz, 1H), 3.71 (td, *J* = 9.1, 8.6, 7.2 Hz, 1H), 3.59 (ddd, *J* = 9.8, 8.0, 4.1 Hz, 1H), 2.37 – 2.24 (m, 1H), 2.22 – 2.07 (m, 1H), 2.05 – 1.89 (m, 1H), 1.91 – 1.74 (m, 1H), 1.46 (d, *J* = 6.8 Hz, 3H), 1.39 (d, *J* = 6.9 Hz, 3H).
LCMS m/z 442.1 $(M-H)^-$

N-(2-((*S*)-2-(((*S*)-1-(4-methoxyphenyl)ethyl)carbamoyl)pyrrolidin-1-yl)-2-oxoethyl)quinoline-2-carboxamide (6)

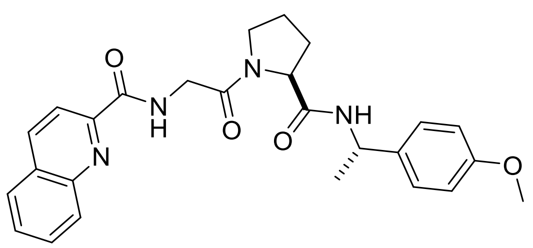

(Quinoline-2-carbonyl)glycyl-L-proline
To a mixture of (quinoline-2-carbonyl)glycine (1.9 g, 8.26 mmol) and L-proline-t-butyl ester (1.71 g, 10.0 mmol) in DCM (15 ml) was added PyBOP (4.5 g, 8.65 mmol) and DIPEA (2.24 ml, 16.5 mmol). The reaction was stirred at rt for 16 hours and then washed with aq. sodium bicarbonate, dried and evaporated. Purification by FC (SiO_2, ethyl acetate/MeOH) afforded the intermediate ester (2.68 g). To a stirred solution of the ester (1.3 g) dissolved in DCM (7 ml) at 0 ˚C was added tri-isopropylsilane (0.1 ml) and then TFA (10 ml). The mixture was stirred for four hours. The solvent was evaporated and the mixture was partitioned between ethyl acetate and 0.8 M aq HCl. The organics were separated, dried and evaporated to generate the product (800 mg, 61% over two steps).
^{1}H NMR (400 MHz, Chloroform-*d*) δ 9.33 (s, 0.2H), 9.05 – 8.96 (m, 0.8H), 8.37 – 8.23 (m, 2H), 8.22 – 8.14 (m, 1H), 7.94 – 7.85 (m, 1H), 7.79 (ddd, *J* = 8.5, 6.9, 1.5 Hz, 1H), 7.69 – 7.60 (m, 1H), 4.76 – 4.67 (m, 0.8H), 4.63 – 4.56 (m, 0.2H), 4.54 – 4.24 (m, 2H), 3.86 – 3.53 (m, 2H), 2.49 – 1.91 (m, 4H).
LCMS m/z 328.1 $(M+H)^+$

***N*-(2-((*S*)-2-(((*S*)-1-(4-methoxyphenyl)ethyl)carbamoyl)pyrrolidin-1-yl)-2-oxoethyl)quinoline-2-carboxamide (6)**

Methoxy-(*S*)-α-methylbenzylamine (45 µL, 0.31 mmol) was coupled to (quinoline-2-carbonyl)glycyl-*L*-proline (50 mg, 0.15 mmol) according to the general procedure A to give the product as a clear oil (39 mg, 55%).
^{1}H NMR (400 MHz, Chloroform-*d*) δ 9.01 – 8.90 (m, 1H), 8.31 (d, *J* = 8.4 Hz, 1H), 8.25 (d, *J* = 8.4 Hz, 1H), 8.19 (dq, *J* = 8.6, 0.9 Hz, 1H), 7.90 (dd, *J* = 8.1, 1.5 Hz, 1H), 7.79 (ddd, *J* = 8.4, 6.9, 1.5 Hz, 1H), 7.65 (ddd, *J* = 8.2, 6.9, 1.2 Hz, 1H), 7.28 – 7.23 (m, 2H), 7.18 (d, *J* = 7.8 Hz, 1H), 6.86 – 6.79 (m, 2H), 5.03 (p, *J* = 7.1 Hz, 1H), 4.62 (dd, *J* = 8.2, 2.2 Hz, 1H), 4.35 (t, *J* = 4.8 Hz, 2H), 3.73 (s, 4H), 3.57 (td, *J* = 9.4, 7.0 Hz, 1H), 2.45 – 2.36 (m, 1H), 2.31 – 2.16 (m, 1H), 2.12 – 2.02 (m, 1H), 1.99 – 1.87 (m, 1H), 1.49 (d, *J* = 7.0 Hz, 3H).
LCMS *m/z* 461.4 $(M+H)^+$

N-(2-((2*S*,4*R*)-2-(((*S*)-1-(2-chloro-4-methoxyphenyl)ethyl)carbamoyl)-4-hydroxypyrrolidin-1-yl)-2-oxoethyl)-6-fluoroquinoline-2-carboxamide CAM833A

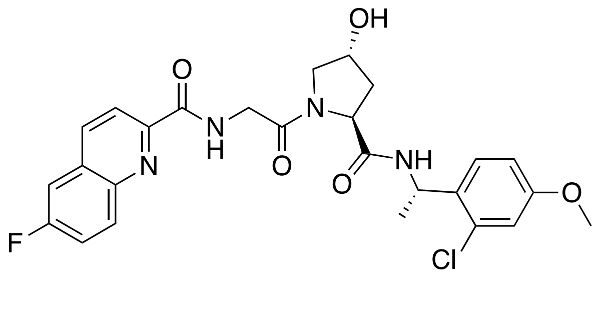

(*S*)-1-(2-Chloro-4-methoxyphenyl)ethan-1-amine

2-Chloro-4-methoxybenzyaldehyde (3.4 g, 19.9 mmol) and (*R*)-(+)-t-butanesulfinamide (2.4 g, 19.9 mmol) were added to THF (100 mL) and $Ti(OEt)_4$ (10.0 g, 43.8 mmol) was added. The reaction was heated at reflux overnight under N_2, cooled to room temperature and brine (100 mL) and ethyl acetate (100 mL) added. The mixture was filtered through celite, washing the celite with ethyl acetate (100 mL). The separated organic layer was dried (Na_2SO_4), filtered, and the solvent removed in vacuo to give the sulfinimide as a white solid (4.9 g, 90%). LCMS *m/z* 274.2 $(M+H)^+$

A portion of sulfinimide (2.5 g, 9.1 mmol) was dissolved in DCM (60 mL) and cooled to -50 ˚C. MeMgBr (6.0 mL, 3.2 M in 2-methyl THF, 19.2 mmol) was added dropwise and the reaction mixture allowed to warm to rt overnight. Saturated aq. NH_4Cl (50 mL) was added, mixture was stirred for 5 mins and extracted with DCM (2 x 50 mL). The combined organic layers were dried through a hydrophobic frit and purified by FC (SiO_2, 12-100% ethyl acetate:pet ether 40-60) to give the sulfinamine as a clear oil (2.5 g, 94%). The diastereomeric ratio following the Grignard addition step was 98:2 as determined by ^{1}H NMR.

^{1}H NMR (400 MHz, Chloroform-*d*) δ 7.31 (d, *J* = 8.7 Hz, 1H), 6.89 (d, *J* = 2.6 Hz, 1H), 6.80 (dd, *J* = 8.6, 2.6 Hz, 1H), 4.96 (qd, *J* = 6.7, 4.3 Hz, 1H), 3.78 (s, 3H), 1.52 (d, *J* = 6.7 Hz, 3H), 1.19 (s, 9H).

The sulfinamine (2.5 g, 8.6 mmol) was dissolved in 1,4-dioxane (60 mL) and HCl (8 mL, 4N in 1,4-dioxane) was added dropwise. After stirring for 1 hour at room temperature, the solvent was removed *in vacuo*, water (40 mL) and DCM (40 mL) were added and the organic layer discarded. The pH of the aqueous layer was adjusted to ~14 with NaOH pellets, extracted with DCM (2 x 50 mL) and the solvent removed to give the product amine as a clear oil (1.6 g, 100%).

^{1}H NMR (400 MHz, Chloroform-*d*) δ 7.35 (d, *J* = 8.7 Hz, 1H), 6.82 (d, *J* = 2.6 Hz, 1H), 6.75 (dd, *J* = 8.6, 2.6 Hz, 1H), 4.45 – 4.38 (m, 1H), 3.72 (s, 3H), 1.30 (d, *J* = 6.6 Hz, 3H).

(6-Fluoroquinoline-2-carbonyl)glycine

6-Fluoroquinoline-2-carboxylic acid (7 g, 37 mmol) and glycine methyl ester hydrochloride (5.1 g, 40 mmol) were dissolved in DCM (180 mL) and DIPEA (14.8 mL, 80.5 mmol) and cooled to 0 ˚C. Over 30 minutes PyBOP (21 g, 40 mmol) was added portionwise to the solution and stirring continued for 16 h allowing the reaction to warm to rt. The yellow solution was then concentrated *in vacuo* and the resulting oil diluted in ethyl acetate (300 mL). The organic solution was then washed with water (4 x 100 mL), dried over $MgSO_4$ and purified by FC (SiO_2). The resultant methyl ester was dissolved in a MeOH, THF, water mix (1:2:1; 120 mL) and lithium hydroxide added. The solution was stirred for 1h before concentrating in vacuo and acidifying with HCl (3M aqueous). The suspension was then extracted with ethyl acetate (3 x 150 mL) and the combined organics dried and concentrated to give product (7.6 g, 84%) as a white solid.

^{1}H NMR (400 MHz, Chloroform-*d*) δ 8.65 – 8.57 (m, 1H), 8.30 (dd, *J* = 8.5, 0.8 Hz, 1H), 8.25 (dd, *J* = 8.6, 0.8 Hz, 1H), 8.14 (ddt, *J* = 9.3, 5.5, 0.7 Hz, 1H), 7.54 (ddd, *J* = 9.2, 8.2, 2.8 Hz, 1H), 7.48 (dd, *J* = 8.7, 2.8 Hz, 1H), 4.24 (d, *J* = 5.5 Hz, 2H).

LCMS *m/z* 249.0 $(M+H)^+$

Benzyl (2*S*,4*R*)-1-((6-fluoroquinoline-2-carbonyl)glycyl)-4-hydroxypyrrolidine-2-carboxylate
Prepared according to general procedure B using (6-fluoroquinoline-2-carbonyl)glycine (900 mg, 3.6 mmol), benzyl (2*S*,4*R*)-4-hydroxypyrrolidine-2-carboxylate (1 g, 4.0 mmol), DIPEA (2.4 mL, 18 mmol), DCM (20 mL) and PyBOP (1.9 g, 4.0 mmol), purified by FC (SiO_2, 2-10% MeOH in DCM) to give the product (1.6 g, 98%) as a white solid.
^{1}H NMR (400 MHz, Chloroform-*d*) δ 8.99 – 8.85 (m, 1H), 8.31 – 8.20 (m, 2H), 8.21 – 8.12 (m, 1H), 7.54 (ddd, *J* = 9.3, 8.2, 2.8 Hz, 1H), 7.48 (dd, *J* = 8.7, 2.7 Hz, 1H), 7.43 – 7.26 (m, 5H), 5.29 – 5.13 (m, 2H), 4.76 (t, *J* = 8.0 Hz, 1H), 4.64-4.61 (m, 1H), 4.44 (d, *J* = 4.8 Hz, 0.4H), 4.39 (d, *J* = 4.8 Hz, 0.6H), 4.28 (d, *J* = 4.5 Hz, 0.6H), 4.24 (d, *J* = 4.5 Hz, 0.4H), 3.80 (d, *J* = 4.4 Hz, 0.4H), 3.78 (d, *J* = 4.3 Hz, 0.6H), 3.71 – 3.60 (m, 1H), 2.42-2.36 (m, 1H), 2.09 (ddd, *J* = 13.3, 8.0, 4.8 Hz, 2H).

(2*S*,4*R*)-1-((6-fluoroquinoline-2-carbonyl)glycyl)-4-hydroxypyrrolidine-2-carboxylic acid
To a solution of benzyl (2*S*,4*R*)-1-((6-fluoroquinoline-2-carbonyl)glycyl)-4-hydroxypyrrolidine-2-carboxylate (1.1g, 2.45 mmol) in MeOH (24 mL) was added palladium (10% activated on charcoal; 100 mg). The resultant mixture was hydrogenated for 16 h before filtering through celite (MeOH eluent) and concentrating *in vacuo*. Purification by FC (SiO_2; 2-20% MeOH in DCM + 1% acetic acid) gave the title compound (850 mg, 97%) as a white solid.
^{1}H NMR (400 MHz, Chloroform-*d*) δ 8.90 (q, *J* = 6.8, 5.8 Hz, 1H), 8.15 (d, *J* = 8.6 Hz, 1H), 8.08 (dd, *J* = 8.6, 0.8 Hz, 1H), 8.01 (dd, *J* = 9.3, 5.2 Hz, 1H), 7.46 – 7.33 (m, 2H), 4.56 – 4.44 (m, 1H), 4.46 – 4.37 (m, 1H), 4.30 – 4.18 (m, 1H), 4.16 – 4.05 (m, 1H), 3.69 – 3.58 (m, 1H), 3.51 – 3.41 (m, 1H), 2.25 – 2.15 (m, 1H), 2.06 – 1.94 (m, 1H), 1.36 – 1.17 (m, 1H).
LCMS *m/z* 362.2 $(M+H)^+$

***N*-(2-((2*S*,4*R*)-2-(((*S*)-1-(2-chloro-4-methoxyphenyl)ethyl)carbamoyl)-4-hydroxypyrrolidin-1-yl)-2-oxoethyl)-6-fluoroquinoline-2-carboxamide CAM833A**
(2*S*,4*R*)-1-((6-fluoroquinoline-2-carbonyl)glycyl)-4-hydroxypyrrolidine-2-carboxylic acid (3.6 g, 10.1 mmol) and (*S*)-1-(2-chloro-4-methoxyphenyl)ethan-1-amine (1.6 g, 8.61 mmol) were dissolved in DCM (50 mL), DIPEA (8.0 mL, 45.9 mmol) was added and the reaction mixture was cooled to 0 ˚C. HBTU (4.0 g. 10.6 mmol) was added portion-wise over 30 mins and the reaction mixture was allowed to warm to room temperature and stirred for 3 hours. The resulting precipitate was removed by filtration, and dissolved in DCM (200 mL) and washed with water (200 mL). Some precipitation occurred during the aqueous wash, which was collected by filtration, dissolved in ethyl acetate (150 mL) and washed with water (2 x 100 mL). The DCM organic layer was concentrated, and ethyl acetate (150 mL) was added and washed with water (2 x 100 mL). The organic layers were combined, dried with brine and magnesium sulfate and the solvent removed *in vacuo* to give the product as a white solid (2.8 g, 61%).
^{1}H NMR (500 MHz, DMSO-*d6*) δ 9.00-8.94 (m, 1H), 8.83 (d, J = 7.5 Hz, 0.3H), 8.58 (d, J = 8.5 Hz, 1H), 8.44 (d, J = 7.5 Hz, 0.7H), 8.25-8.18 (m, 2H), 7.93 (dd, J = 9.0, 2.5 Hz, 1H), 7.82 (m, 1H), 7.36 (d, J = 8.5 Hz, 0.3H), 7.29 (d, J = 8.5 Hz, 0.7H), 7.00 (d, J = 2.5 Hz, 0.3H), 6.95 (d, J = 2.5 Hz, 0.7H), 6.94 (dd, J = 2.5 Hz, 0.3H), 6.86 (dd, J = 8.5 Hz, 2.5 Hz, 0.7H), 5.19 (m, 1H), 5.08 (m, 0.7H), 4.60 (t, J = 7.5 Hz, 0.3H), 4.42 (t, J = 7.5 Hz, 0.7H), 4.35 (m, 0.7H), 4.27-4.12 (m, 2H), 3.87 (dd, 17.0, 5.5 Hz, 0.3H), 3.75 (s, 0.9H), 3.71 (s, 2.1H), 3.70-3.39 (m, 2H), 2.25 (m, 0.3H), 2.06 (m, 0.7H), 1.94 (m, 0.3H), 1.78 (m, 0.7H), 1.39 (d, J = 7.0 Hz, 0.9H), 1.30 (d, J = 7.0 Hz, 2.1H).
LCMS *m/z* 529.3 $(M+H)^+$
