# Supplementary material for: A small-molecule inhibitor of the BRCA2-RAD51 interaction modulates RAD51 assembly and potentiates DNA damage-induced cell death
Source: Cell Chem Biol. 2021 Jun 17;28(6):835–847.e5. doi: 10.1016/j.chembiol.2021.02.006 (PMC8219027; doi:10.1016/j.chembiol.2021.02.006)
Supplement: Document S2. Article plus Supplemental information [file mmc2.pdf]

Article

Cell Chemical Biology

A small-molecule inhibitor of the BRCA2-RAD51 interaction modulates RAD51 assembly and potentiates DNA damage-induced cell death

Graphical abstract

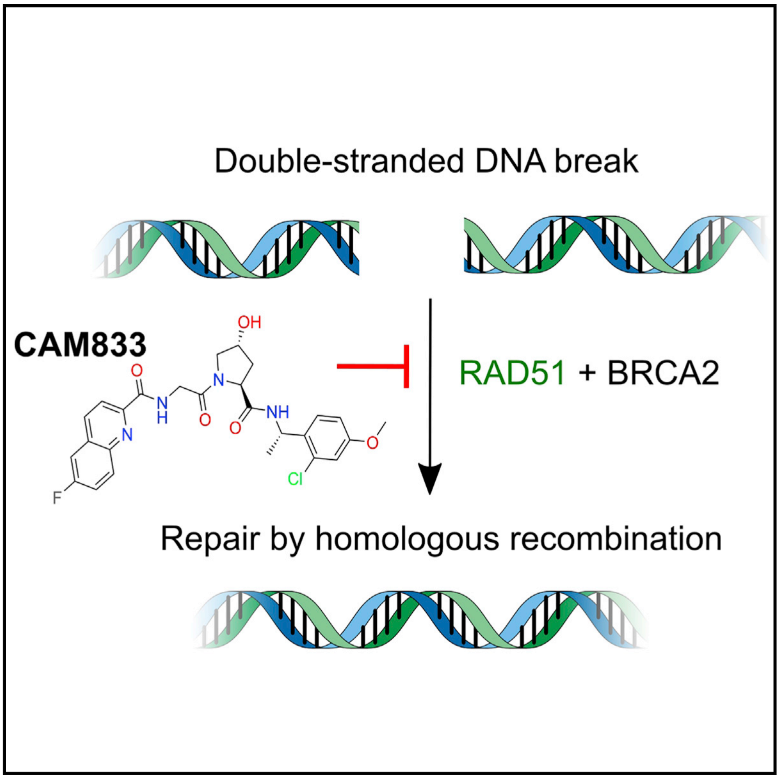

Authors

Duncan E. Scott,
Nicola J. Francis-Newton,
May E. Marsh, ..., John Skidmore,
Ashok R. Venkitaraman, Chris Abell

Correspondence

mh256@cam.ac.uk (M.H.),
js930@cam.ac.uk (J.S.),
arv22@nus.edu.sg (A.R.V.)

In Brief

Scott et al. describe CAM833, an inhibitor of DNA recombinase RAD51, that binds at the site normally occupied by the BRC repeats of the BRCA2 protein. Through blocking this protein-protein interaction, CAM833 is able to prevent RAD51-mediated homologous recombination and thus potentiate cancer cells to damage by radiation or PARP1 inhibition.

Highlights

- CAM833 inhibits the protein-protein interaction between RAD51 and BRCA2
- Crystal structure shows CAM833 binds RAD51 at the same site as the BRCA2 FxxA motif
- CAM833 blocks formation of RAD51 foci and filaments, preventing DNA repair
- CAM833 potentiates cytotoxicity by IR and synergises with PARP1 inhibitors

Scott et al., 2021, Cell Chemical Biology *28*, 835–847
June 17, 2021 © 2021 The Authors. Published by Elsevier Ltd.
https://doi.org/10.1016/j.chembiol.2021.02.006

CellPress

Cell Chemical Biology

CellPress
OPEN ACCESS

Article

A small-molecule inhibitor of the BRCA2-RAD51 interaction modulates RAD51 assembly and potentiates DNA damage-induced cell death

Duncan E. Scott,[1,5,21] Nicola J. Francis-Newton,[2,21] May E. Marsh,[3,6] Anthony G. Coyne,[1] Gerhard Fischer,[3,7] Tommaso Moschetti,[3,8] Andrew R. Bayly,[1,9] Timothy D. Sharpe,[3,10] Kalina T. Haas,[2,15] Lorraine Barber,[2] Chiara R. Valenzano,[1,11] Rajavel Srinivasan,[1,12,19] David J. Huggins,[1,2,13] Miyoung Lee,[2] Amy Emery,[2] Bryn Hardwick,[2] Matthias Ehebauer,[3,14] Claudio Dagostin,[1,20] Alessandro Esposito,[2] Luca Pellegrini,[3] Trevor Perrior,[4] Grahame McKenzie,[2,16] Tom L. Blundell,[3] Marko Hyvönen,[3,*] John Skidmore,[1,5,23,*] Ashok R. Venkitaraman,[2,17,18,*] and Chris Abell[1,22]

[1]Yusuf Hamied Department of Chemistry, University of Cambridge, Lensfield Road, Cambridge CB2 1EW, UK
[2]Medical Research Council Cancer Unit, University of Cambridge, Hutchison/MRC Research Centre, Hills Road, Cambridge CB2 0XZ, UK
[3]Department of Biochemistry, University of Cambridge, 80 Tennis Court Road, Cambridge CB2 1GA, UK
[4]Excellium Consulting, Brook Farm Barn, Lackford, Bury St Edmunds IP28 6HL, UK
[5]Present address: The ALBORADA Drug Discovery Institute, University of Cambridge, Island Research Building, Hills Road, Cambridge CB2 0AH, UK
[6]Present address: Paul Scherrer Institut, Villigen PSI, Switzerland
[7]Present address: Boehringer Ingelheim RCV, Doktor-Boehringer-Gasse 5-11, 1121 Vienna, Austria
[8]Present address: Illumina Cambridge Ltd, Illumina Center, Granta Park, Great Abington, Cambridge CB21 6DF, UK
[9]Present address: Vertex Pharmaceuticals Limited, 86-88 Jubilee Avenue, Milton Park, Abingdon, Oxfordshire OX14 4RW, UK
[10]Present address: Biophysics Facility, Biozentrum, University of Basel, Basel, Switzerland
[11]Present address: Astex Pharmaceuticals, 436 Cambridge Science Park, Milton Road, Cambridge CB4 0QA, UK
[12]Present address: School of Pharmaceutical Science and Technology, Health Science Platform, Tianjin University, 92 Weijin Road, Nankai District, Tianjin 300072, People's Republic of China
[13]Present address: Department of Physiology and Biophysics, Weill Cornell Medical College, New York, NY, USA
[14]Present address: Ipsen Bioinnovation, Oxford, UK
[15]Present address: Institut Jean-Pierre Bourgin, INRAE, AgroParisTech, Université Paris-Saclay, 78000 Versailles, France
[16]Present address: PhoreMost Ltd., Babraham Research Campus, Cambridge CB22 3AT, UK
[17]Present address: The Cancer Science Institute of Singapore, Center for Translational Medicine, National University of Singapore, Singapore 117599, Singapore
[18]Present address: Agency for Science, Technology and Research (A*STAR), 8A Biomedical Grove, Singapore 138648, Singapore
[19]Present address: Singapore Eye Research Institute, The Academia, 20 College Road, Discovery Tower, Singapore 169856, Singapore
[20]Present address: o2h group, Mill SciTech Park, Mill Lane Hauxton, Cambridge CB22 5HX, UK
[21]These authors contributed equally
[22]Prof Chris Abell died on October 26, 2020, during the revision period for this manuscript. The authors dedicate this paper to their good friend and colleague.
[23]Lead contact
*Correspondence: mh256@cam.ac.uk (M.H.), js930@cam.ac.uk (J.S.), arv22@nus.edu.sg (A.R.V.)
https://doi.org/10.1016/j.chembiol.2021.02.006

SUMMARY

BRCA2 controls RAD51 recombinase during homologous DNA recombination (HDR) through eight evolutionarily conserved BRC repeats, which individually engage RAD51 via the motif Phe-x-x-Ala. Using structure-guided molecular design, templated on a monomeric thermostable chimera between human RAD51 and archaeal RadA, we identify CAM833, a 529 Da orthosteric inhibitor of RAD51:BRC with a K_d of 366 nM. The quinoline of CAM833 occupies a hotspot, the Phe-binding pocket on RAD51 and the methyl of the substituted α-methylbenzyl group occupies the Ala-binding pocket. In cells, CAM833 diminishes formation of damage-induced RAD51 nuclear foci; inhibits RAD51 molecular clustering, suppressing extended RAD51 filament assembly; potentiates cytotoxicity by ionizing radiation, augmenting *4N* cell-cycle arrest and apoptotic cell death and works with poly-ADP ribose polymerase (PARP)1 inhibitors to suppress growth in BRCA2-wildtype cells. Thus, chemical inhibition of the protein-protein interaction between BRCA2 and RAD51 disrupts HDR and potentiates DNA damage-induced cell death, with implications for cancer therapy.

This is an open access article under the CC BY license (http://creativecommons.org/licenses/by/4.0/).

INTRODUCTION

The tumor suppressor protein, BRCA2, is essential for error-free repair of DNA double-stranded breaks (DSBs) by homologous DNA recombination (HDR) in human cells (Venkitaraman, 2014). BRCA2 acts during HDR to control the recombination enzyme, RAD51, a eukaryal protein evolutionarily conserved as RecA in eubacteria, and RADA in archaea (West, 2003). RAD51 executes the DNA strand exchange reactions that lead to HDR by assembling, in a sequential and highly regulated manner, as helical nucleoprotein filaments on single-stranded (ss) or double-stranded (ds) DNA substrates. The presynaptic RAD51 filament on ssDNA mediates strand invasion and homologous pairing with a duplex DNA template to execute strand exchange, the core biochemical event necessary for HDR.

Human BRCA2 contains two distinct regions that bind directly to RAD51. First, BRCA2 contains eight BRC repeats, evolutionarily conserved motifs of 26 residues each, whose sequence and spacing within a ~1,100-residue segment encoded by *BRCA2* exon 11 is conserved among mammalian orthologues (Bignell et al., 1997). Each of the eight human BRC repeats exhibits a varying affinity for RAD51 *in vitro* (Wong et al., 1997). Second, the carboxyl (C-) terminus of BRCA2 contains a RAD51-binding region spanning ~90 residues, which is distinct in sequence from the BRC repeats (Davies and Pellegrini, 2007; Esashi et al., 2007).

The interactions between BRCA2 and RAD51 control key steps essential for HDR. The BRC repeat-RAD51 interaction differentially regulates RAD51 assembly on DNA substrates *in vitro*, promoting assembly of the RAD51-ssDNA filament, while concurrently inhibiting the RAD51-dsDNA interaction (Carreira et al., 2009; Shivji et al., 2009). These opposing activities of the BRC repeats ensure that RAD51 assembly on its DNA substrates occurs in the correct order to promote strand exchange. Moreover, the C-terminal RAD51-binding region of BRCA2 stabilizes oligomeric assemblies of RAD51 *in vitro* in biochemical assays using purified proteins (Davies and Pellegrini, 2007; Esashi et al., 2007), and is required for the elongation of RAD51 filaments at cellular sites of DNA damage visualized by single-molecule localization microscopy (Haas et al., 2018).

Of the eight BRC repeats in human BRCA2, BRC4 exhibits the highest affinity for RAD51 (Carreira and Kowalczykowski, 2011; Cole et al., 2017; Wong et al., 1997). The crystallographic structure of a complex between a BRC4 peptide and the core catalytic domain of RAD51 shows that the BRC4 sequence FHTA (human BRCA2 residues 1524–1527) engages with hydrophobic pockets on the RAD51 surface that accommodate the Phe and Ala residues (Pellegrini et al., 2002). An analogous FxxA motif in the RAD51 protein mediates oligomerization in the absence of DNA (Brouwer et al., 2018; Conway et al., 2004; Shin et al., 2003), and has recently been shown using electron cryo-microscopy to form the inter-subunit interface in functionally relevant DNA-bound assemblies of RAD51 (Short et al., 2016; Xu et al., 2017). *In vitro*, BRC4 peptides promote the strand selectivity of RAD51-DNA interactions at sub-stoichiometric concentrations relative to RAD51 (Carreira et al., 2009; Shivji et al., 2009). However, BRC4 peptides disrupt RAD51 oligomerization *in vitro* (Davies et al., 2001), and when overexpressed in cells, can inhibit the recruitment of RAD51 into DNA damage-induced foci by blocking the RAD51-RAD51 interaction (Chen et al., 1999a).

The central importance of the BRC repeat–RAD51 interaction to HDR has prompted the identification of small-molecule and peptidic inhibitors that might have therapeutic value for cancer treatment. Most reported inhibitors target the interaction between RAD51 and DNA (Budke et al., 2012a, 2012b; Huang and Mazin, 2014; Huang et al., 2011; Ishida et al., 2009; Normand et al., 2014; Takaku et al., 2011). Recently described cell-penetrating antibodies also operate through a similar mechanism (Pastushok et al., 2019; Turchick et al., 2017, 2019). Inhibitors that suppress the D-loop activity of RAD51 have also been reported (Budke et al., 2019; Lv et al., 2016), although several optimized versions also exhibit DNA-intercalating activity (Budke et al., 2019). On the other hand, reports of small molecules and peptides have been published that claim to disrupt the interaction between RAD51 and the BRC repeats, or between RAD51 multimers (Bagnolini et al., 2020; Falchi et al., 2017; Nomme et al., 2010; Roberti et al., 2019; Trenner et al., 2018; Vydyam et al., 2019; Zhu et al., 2013, 2015; Ward et al., 2017). However, the lack of specific structural information concerning the interaction of these inhibitors with RAD51 has impeded the precise exploration of structure-activity relationships, and the efficient development of more potent compounds.

Here, we report the discovery, using a structure-led fragment-based approach, of CAM833, a potent chemical inhibitor of the RAD51-BRC repeat interaction and RAD51 oligomerization. We show using X-ray crystallography that CAM833 engages the Phe- and Ala-binding pockets on RAD51 to block its interaction with BRC repeats. We confirm that CAM833 potentiates cellular sensitivity to DNA damage induced by ionizing radiation, and suppresses the assembly of RAD51 into damage-induced filaments, as visualized by single-molecule localization microscopy. Our findings provide a well-characterized chemical tool compound to dissect biochemical events during HDR, and a potential lead for the development of new cancer therapeutics.

RESULTS

A monomeric thermostable chimera of human RAD51 and archaeal RADA recapitulates structural features of the human RAD51-BRC interaction

Structure-based approaches to identify modulators of the BRCA2–RAD51 interaction have been impeded by the lack of a monomeric unliganded form of *Hs*RAD51. We have previously described the development of molecular surrogate systems for RAD51 based on an archaeal ortholog, RadA from *Pyrococcus furiosus* (Moschetti et al., 2016). In brief, we were able to produce the C-terminal ATPase domain of RadA as a stable monomeric protein, and by careful mutagenesis, to convert the surface of the protein to resemble human RAD51, with the ability to bind the BRCA2 BRC4 repeat with high affinity. Of note, we used the previously described constructs HumRadA2 for initial biophysics work and HumRadA22F for crystallography (Moschetti et al., 2016). In parallel, we also generated a chimeric RAD51 (ChimRAD51) that fuses the central part of the human RAD51 ATPase domain with two flanking sequences from archaeal RadA and used this in our primary screening assay and for biophysical screening. The binding of ChimRAD51 to the BRC4 peptide was characterized using a

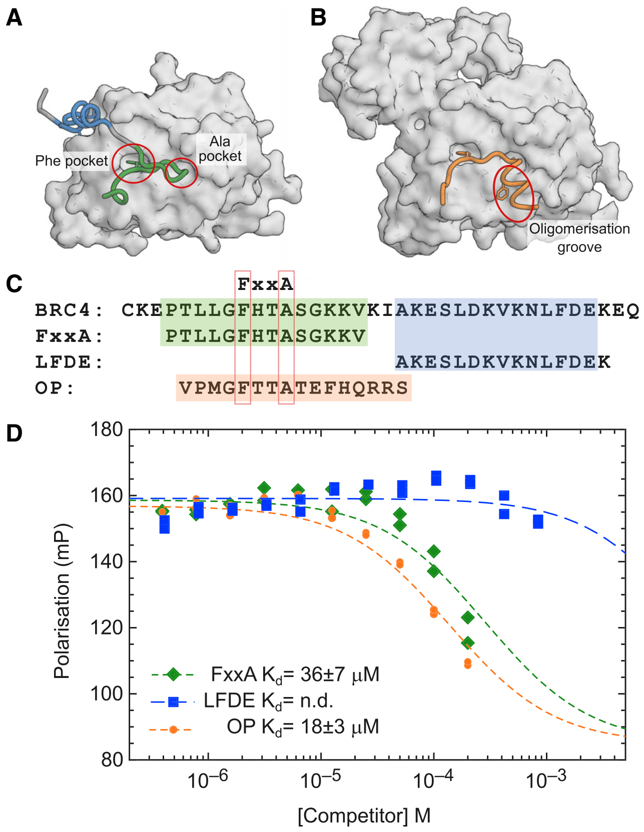

Figure 1. RAD51 interaction with BRC4

(A) Structure of RAD51 ATPase domain (surface) with BRC4 repeat of BRCA2 with FxxA binding motif colored green and the LFDE-motif in blue (PDB: 1n0w).

(B) Structure of oligomeric RAD51 with oligomerization epitope (orange) of one protomer binding the next molecule in the filament (surface) (PDB: 5nwl).

(C) Sequences of BRC4 repeat, and its FxxA and LFDE epitopes containing half peptides and the isolated RAD51 oligomerization peptide (OP).

(D) Competitive FP assay with labeled BRC4 repeat as probe which shows competitive binding to ChimRAD51 protein with the two BRC4 half-peptides (FxxA and LFDE, green and blue) and RAD51 oligomerization peptide (OP, orange). The dissociation constants measured for the FxxA half-peptide and for the oligomerization peptides were 36 ± 7 μM and 18 ± 3 μM, respectively.

fluorescence polarization (FP) assay and by isothermal titration calorimetry (ITC), yielding comparable K_d values of 4 and 6 nM, respectively validating the use of this protein for subsequent ligand affinity measurements (Moschetti et al., 2016). These surrogates provide robust platforms for structure-guided lead discovery via fragment screening, the biophysical characterization and validation of inhibitors, and for X-ray crystallography.

The three-dimensional structure of the C-terminal ATPase domain of RAD51 in complex with a BRC4 peptide has been determined by X-ray crystallography (Pellegrini et al., 2002). This structure shows that the BRC repeat binds in a bidentate fashion in which BRC4, via its FxxA motif, engages with a self-association site on RAD51, and then wraps around the protein to interact through a less-conserved LFDE motif with a second site on the RAD51 surface (Figure 1A). Cryo-EM structures of RAD51 filaments bound to DNA (Short et al., 2016; Xu et al., 2017) confirm that in self-association, the FxxA motif of one RAD51 interacts similarly with the two small "Phe" and "Ala" pockets on an adjacent protein unit, with the C-terminal segment of the oligomerization epitope binding to a hydrophobic groove in the opposite direction to that where the LFDE epitope of BRC4 binds. Earlier work has compared the relative affinities of the different human BRC repeats for RAD51 (e.g., Wong et al., 1997), and has demonstrated that both the FxxA and LFDE motifs in multiple BRC repeats contribute to both permissive and inhibitory interactions with RAD51 (Rajendra and Venkitaraman, 2010). In order to determine which of these two motifs might be most appropriate to develop inhibitors against, we measured the affinities of two peptides corresponding to N- and C-terminal epitopes of BRC4 using our FP assay. The N-terminal "FxxA" half of the BRC4 repeat showed clear binding to RAD51 and competition of full-length BRC4 repeat with a K_d of 36 μM. This compares favorably with our previous analysis of the affinities of tetra-peptides derived from the BRC4 FxxA epitope (which has the sequence FHTA), which bound to humanized RadA with 200 to 300 μM affinity (Scott et al., 2016). The C-terminal half of BRC4 (LFDE peptide) showed very weak, if any, inhibition of BRC4 binding, at up to 1 mM concentration (Figure 1C), suggesting that the LFDE motif makes a minimal contribution on its own to this interaction, even though its mutation in the context of the entire BRC4 peptide can reduce RAD51 binding (Rajendra and Venkitaraman, 2010). We also tested the ability of RAD51 to bind its own oligomerization peptide (OP) epitope and determined a K_d of 18 μM for this interaction, demonstrating how additional binding energy can be derived from the interactions that the C-terminal part of the oligomerization peptide makes (Figure 1C).

The design and development of CAM833, a small molecule inhibitor of the interaction between BRCA2 and RAD51

Using the surrogate RAD51 systems described above and a combination of fragment-based drug discovery (Blundell et al., 2002; Coyne et al., 2010) and structure-guided drug design, we have optimized fragment hit molecules to generate high-affinity inhibitors of the RAD51–BRC-repeat interaction with a clearly defined orthosteric inhibition mechanism (Figure 2).

Initially, we carried out a biophysical fragment-screen against a previously described humanized version of *Pf*RadA HumRadA2 (also known as MAYSAM [Moschetti et al., 2016; Scott et al., 2013]), leading to the discovery of a range of bicyclic aromatic and heteroaromatic fragment hits, binding exclusively into the Phe pocket at the FxxA site of RAD51 (Scott et al., 2013). Investigation of the structure-activity relationships (SAR) around these hits showed that naphthyl derivatives, particularly when substituted with polar groups, were able to bind to the Phe pocket with reasonable activity and ligand efficiency. For example, 2-naphthol (**2**) bound to the HumRadA2 protein with a K_d of 460 μM as measured by ITC (Scott et al., 2013), whereas 3-amino-2-naphthoic acid (**3**) (Figure 2A) bound with a K_d of 1.3 mM (Figure S1). Crystallographic analysis of these fragments shows that the naphthyl rings bind in the same orientation as the aromatic side chain of phenylalanine in the FxxA motif of oligomerization peptide or BRC repeats (Figure 2B) (Scott et al., 2016).

In parallel, we explored the SAR of a series of *N*-acetylated tetrapeptides based on the FxxA epitope of BRC4 (Scott et al., 2016). This work established that the Ac-FHTA-NH_2 tetrapeptide

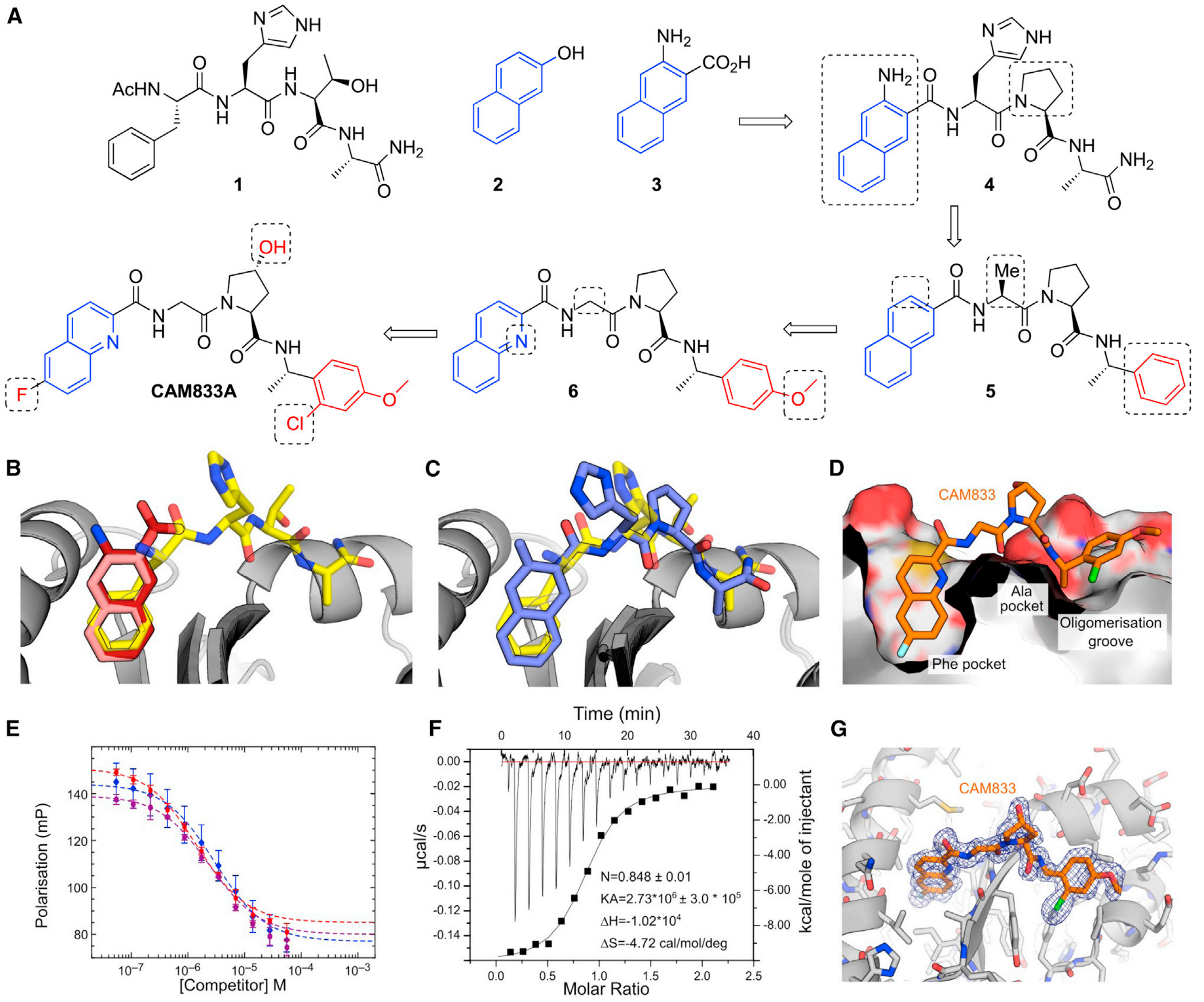

Figure 2. Development of CAM833

(A) Merging of 3-amino-2-naphthoic acid (**3**) with FHPA tetrapeptide to yield **4**. Trimming of the naphthyl and histidine group and replacement of terminal amide with phenyl group yields **5**. Increase of polarity by replacing naphthyl with quinoline and adding methoxy group the phenyl ring results in **6**. Further optimization leads to CAM833.

(B) Overlaid crystal structures of HumRadA1 in complex with 2-naphthol (**2**, PDB: 4B32, pink), 3-amino-2-naphthoic acid (**3**, PDB: 6TV3, dark red) and FHTA tetrapeptide (**1**, PDB: 4B3B, yellow).

(C) Structure of **4** (PDB: 6TWR, deep purple) in complex with HumRadA1 overlaid with FHTA peptide (PDB: 4B3B, yellow).

(D) Structure of CAM833 (orange, PDB: 6TW9) in complex with HumRadA22F. Side view of CAM833 complex with HumRadA22F showing partially cut surface of the protein and interaction of the fluoroquinoline ring with the Phe-pocket and the chloro-phenyl group binding into the oligomerization groove.

(E) Competition of BRC4 peptide binding to ChimRAD51 using FP assay with CAM833. Three independent measurements (triplicate technical repeats) of the same binding are shown in three different colors.

(F) Isothermal titration calorimetric measurement of direct binding of CAM833 to ChimRAD51. The baseline corrected thermogram is shown above with x and y axes above and left of the graph. The solid squares depict integrated heats for each titration point and solid line the fit to single-site binding mode with corresponding x and y axes below and to left of the graph.

(G) Refined $2F_oF_c$ electron density is shown for the ligand, contoured at 1σ.

(**1**) binds to HumRadA2 with a K_d of 280 μM as determined by ITC.

Based on an overlay of the X-ray crystal structures of **1** and the naphthyl fragments 2-naphthol (**2**) and 3-amino-2-naphthoic acid (**3**) (Figure 2B) we designed compound **4** in which the Phe of FHTA has been replaced by a rigid naphthyl-based amino acid, designed to more completely fill this pocket, and the threonine has been replaced by a proline in order to restrict the conformation of the peptide. The latter modification is known to provide a modest potency increase from the tetrapeptide structure activity relationship studies (Scott et al., 2016), with the benefit of removing two H-bond donors from the structure,

a change likely to be associated with an increase in cell permeability. Gratifyingly, the merged compound **4** was found to have improved K_d of 3 μM against HumRadA2 as determined by ITC (Figure S1), a considerable increase in potency compared with the native peptide. We determined an X-ray crystal structure of **4** bound to the HumRadA1 protein and this was found to interact in the predicted fashion, with a modest distortion of the peptide backbone in order to accommodate the more rigid left-hand side (orientation as in Figure 2) (Figure 2C).

Recognizing that the peptidic nature of **4** was likely to lead to poor pharmacokinetics and low permeability (clogP as calculated with ChemDraw 16 -0.96 and tPSA 172 Å^2), we sought to reduce the size and polarity of our compounds while introducing groups capable of forming additional interactions with the protein surface. This led to the design of compound **5,** in which three polar elements judged unnecessary were removed: first, the His residue, which makes no key interactions in the tetrapeptide-protein crystal structure was cut back to an alanine; second, we removed the amino group from the terminal naphthoate unit. Finally, the terminal Ala amide was replaced with an α-methylbenzylamino group that maintains the methyl group important for binding into the Ala pocket while replacing the terminal-amide with a lipophilic phenyl ring, inspired by the relatively non-polar nature of this region of the protein surface. Overall, compound **5** has only two intact amino acids and greatly reduced polarity (clogP 4.08, tPSA = 78.5 Å^2). Compound **5** has a K_d of 220 nM vs HumRadA2 by ITC (Figure S2), a 10-fold potency increase. By this stage, we had developed the more thoroughly humanized form of the protein ChimRAD51, which was subsequently used for our primary FP screening assays (Moschetti et al., 2016). We determined the K_d of **5** against ChimRAD51 to be 1.9 μM by ITC and 27 μM (n = 20) as measured by FP. The reduced level of potency against this more humanized system was mirrored in data with the original 2-naphthol fragment (**2**), which we found to have a K_d of 3.3 mM for ChimRAD51 versus 460 μM for HumRadA2.

Compound **5** was too insoluble in aqueous media to profile in cell-based assays. Accordingly, we made modifications designed to increase polarity, while avoiding the introduction of further hydrogen-bond donors likely to reduce permeability. We replaced the naphthyl ring with a quinoline, converted the Ala residue into a Gly, and introduced a 4-methoxy substituent on the right-hand phenyl ring, leading to **6**. Compound **6** has a clogP of 2.76 and an improved FP K_d of 8.0 μM (n = 22) against ChimRAD51. Two independent X-ray structures of **6** demonstrated that this compound was still bound to the FxxA site with the quinoline accessing the Phe pocket in a similar orientation to the naphthyl in compound **4** albeit with a shifted binding mode discussed in more detail below (Figure S3).

More detailed exploration of the SAR around **6** led to the discovery of **CAM833** with a 6-fluoro substituent on the quinoline and a 2-chloro group on the phenyl leading to a further increase in affinity. **CAM833** has a K_d against the ChimRAD51 protein of 355 nM (n = 8) as measured by FP (Figure 2E) and 366 nM by ITC (Figure 2F). The lipophilicity associated with these groups was balanced by the introduction of a *trans*-4-hydroxyl substituent on the proline ring serving to maintain solubility (clogP of **CAM833** is 2.73, and tPSA 120 Å^2).

As a biochemical test of **CAM833**, we evaluated its ability to disrupt full-length RAD51 oligomers. Using dynamic light scattering, we observe a shift of the average particle size from ~40 nm for oligomeric RAD51 to ~5 nm particles (corresponding closely to the size of a RAD51 monomer) in the presence of excess of CAM833 (Figure S4).

The X-ray crystal structures of **6** and **CAM833** bound to HumRadA22F (the fully humanized RadA surrogate used for crystallography (Moschetti et al., 2016); Figures 2D, 2G, and S3) revealed an altered binding-mode compared to the lead compound **4** (Figure 2C). In this new binding mode, a shift of the backbone of **CAM833** allows the NH of the right-hand benzyl amide to form a hydrogen bond to $Val200_{189}$ (subscript number refers to the equivalent human RAD51 residue, which differs from the surrogate protein residue numbering) via a bridging water-molecule rather than directly to the protein backbone (Figure S5). We attribute this to the truncation of the His residue back to a Gly, a change that can be tracked in the X-ray structures of intermediates from the optimization bound to HumRadA22F (data not shown).

Overall, examination of the structures reveals the source of the potency increases between the tetrapeptide **1** and **CAM833**. The phenyl ring of **CAM833** sits flat on the protein surface with the *ortho*-chlorine atom sitting in a groove leading from this surface with both making beneficial hydrophobic interactions. The quinoline more completely fills the Phe pocket and the 6-fluoro substituent binds into a hydrophobic sub-pocket which has opened up due to minor movements in the residues lining the pocket (Figures 2D and 2G). We determined selectivity and developability data for **CAM833** in order to support its use as a validated chemical probe for the RAD51-BRCA-2 protein-protein interaction. Briefly, **CAM833** is metabolically stable, does not significantly inhibit CYP450 enzymes, shows moderate solubility and permeability, and has no significant off-target interactions when screened at 10 μM in the Cerep ExpresSPanel and has mouse pharmacokinetic data suitable for *in vivo* investigation (Table S1).

CAM833 causes a concentration-dependent decrease in RAD51 foci accompanied by increased DNA damage

The assembly of RAD51 into microscopic foci at cellular sites of DNA damage is competitively inhibited by the overexpression of BRC repeat peptides (Chen et al., 1999a). Indeed, structural studies using X-ray crystallography (Brouwer et al., 2018; Pellegrini et al., 2002; Shin et al., 2003) as well as electron cryomicroscopy (Short et al., 2016; Xu et al., 2017) show that RAD51 assembly is mediated by protomer-protomer contacts that structurally mimic the RAD51-BRC repeat interaction. Because it interrupts these contacts *in vitro*, **CAM833** is expected to suppress the function of RAD51 and prevent the formation of RAD51 foci in cells exposed to DNA damage.

We tested this prediction by monitoring RAD51 foci formation after the exposure of A549 non-small cell lung carcinoma (NSCLC) cells to ionizing radiation (IR), using a robust cell-based assay based on high-content microscopy with the Cellomics ArrayScan V^{TI}, to objectively enumerate RAD51 foci (Jeyasekharan et al., 2013). IR-induced DNA breakage was monitored in the same experiment by enumerating foci containing γH2AX, a phosphorylated form of histone H2AX that is formed at DNA breaks (Rogakou et al., 1998).

Notably, CAM833 inhibited RAD51 foci formation 6 h after exposure to 3 Gy IR, in a concentration-dependent manner

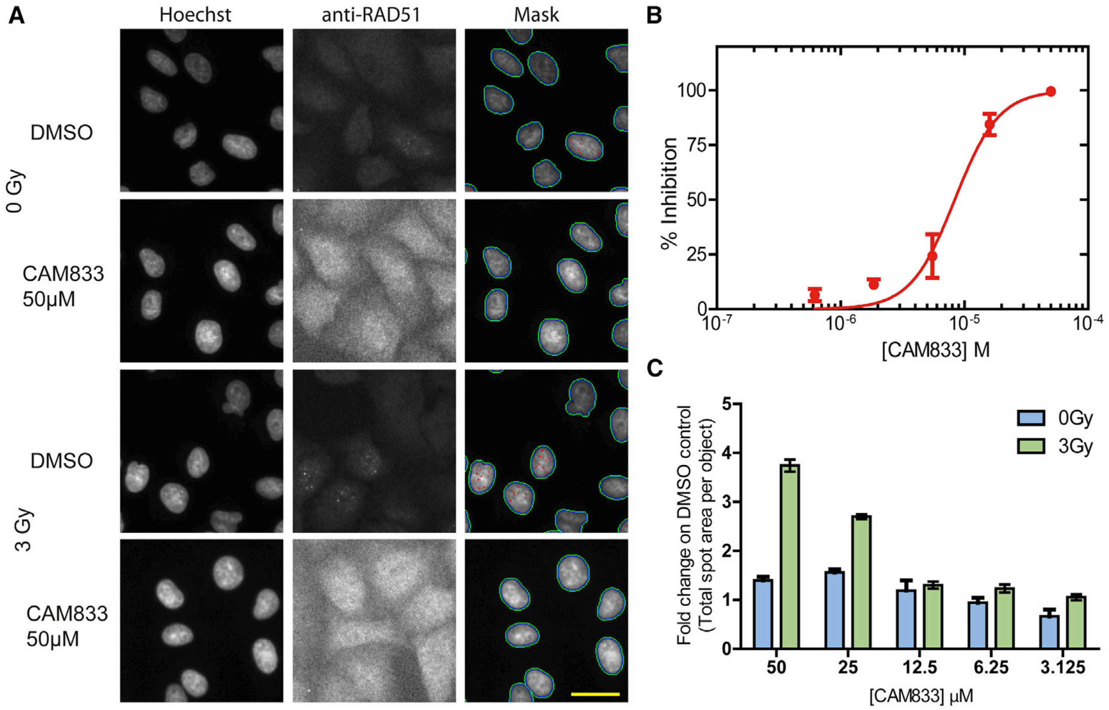

Figure 3. CAM833 causes a concentration-dependent decrease in RAD51 foci and subsequent increase in DNA damage

(A) Images from the Cellomics Arrayscan HCS microscope depicting A549 cells treated with **CAM833** (50 μM) or DMSO controls with or without ionizing radiation (3 Gy). Briefly, cells were co-stained with Hoechst-33342 to identify nuclei and anti-RAD51 antibody to detect RAD51 foci. The final column shows the Hoechst-stained cells with computationally identified nuclei outlined with green, and RAD51 foci with red, respectively. Scale bar, 20 μm (estimated).

(B) An IC_{50} curve calculated from the images collected using the Cellomics HCS microscope as shown in (A). A549 cells were treated with **CAM833**, exposed to 3 Gy ionizing radiation (IR) and fixed after 6 h of incubation. **CAM833** inhibits the formation of IR-induced RAD51 foci in A549 cells with an IC_{50} of 6 μM. Percent inhibition on the y axis was plotted against **CAM833** concentration (as molar) on the x. Plots show mean ± SEM.

(C) Cells treated by the same method were stained and counted for γ-H2AX foci 24 h after exposure. Each pair of bars corresponds to cells exposed to one of five different concentrations (lowest, 3.125 μM on the right, to highest, 50 μM, on the left) of **CAM833** alone (0 Gy), or **CAM833** plus 3 Gy IR (3 Gy). Bars depict the mean values of the fold change in γ-H2AX foci number over control cells treated with DMSO alone, ±SEM. **CAM833** causes a concentration-dependent increase in unresolved DNA damage after 24 h. Results are representative of three independent repeats.

with an IC_{50} of 6 μM (Figures 3A and 3B, plotted as mean ± SEM, n = 27). No RAD51 foci were detected at ~50 μM **CAM833**, corresponding to a maximal level of inhibition. Furthermore, 50 μM **CAM833** increased γH2AX foci formation 24 h after exposure by approximately 4-fold compared with control-treated cells (Figure 3C), suggestive of the persistence of unrepaired DNA damage. These findings are consistent with prior results in cells overexpressing BRC peptides (Chen et al., 1999b), and provide evidence that CAM833 engages its target in the cellular milieu to suppress RAD51 assembly and inhibit DNA repair by HDR.

CAM833 inhibits RAD51 molecular clustering after DNA damage

We have recently visualized the assembly of RAD51 molecules on DNA substrates at cellular sites of DNA damage using single-molecule localization microscopy (SMLM) by direct stochastic optical reconstruction (d-STORM) (Haas et al., 2018). Clusters of approximately 5 to 10 RAD51 molecules are first recruited to DNA damage sites 0.5 to 1 h after damage induction, which progressively extend into filaments >200 nm in length 3 to 5 h afterwards. SMLM shows that RAD51 clustering is suppressed by the overexpression of BRC repeat peptides, indicative of its dependence on protomer-protomer contacts that structurally mimic the RAD51-BRC repeat interaction inhibited *in vitro* by **CAM833**.

Therefore, to test the effect of **CAM833** on RAD51 clustering, we used SMLM on patient-derived EUFA423 cells (Figure 4A) bearing compound heterozygosity for the cancer-associated *BRCA2* truncating alleles 7691*ins*AT and 9000*ins*A (Haas et al., 2018; Howlett et al., 2002). We developed, as isogenic controls, EUFA423 cells complemented by the expression of full-length BRCA2 (EUFA423 + BRCA2) (Hattori et al., 2011). We enumerated the number of RAD51 molecules detected by SMLM in clusters induced by the exposure of EUFA423 cells or EUFA423 + BRCA2 controls (Figure 4A) to 3 Gy IR, in the presence or absence of 25 μM **CAM833**, using a suite of bespoke image analysis algorithms that we have recently reported (Haas et al., 2018). As expected, the accumulation of RAD51 molecules in damage-induced clusters is significantly reduced in BRCA2-deficient EUFA423 cells compared with EUFA423 + BRCA2 controls (Figure 4B) (Hattori et al., 2011). Notably, addition of 25 μM **CAM833** significantly reduces RAD51 accumulation in damage-induced foci to a further extent in both cell types, providing additional evidence that **CAM833** inhibits RAD51 protomer-protomer contacts during filament assembly.

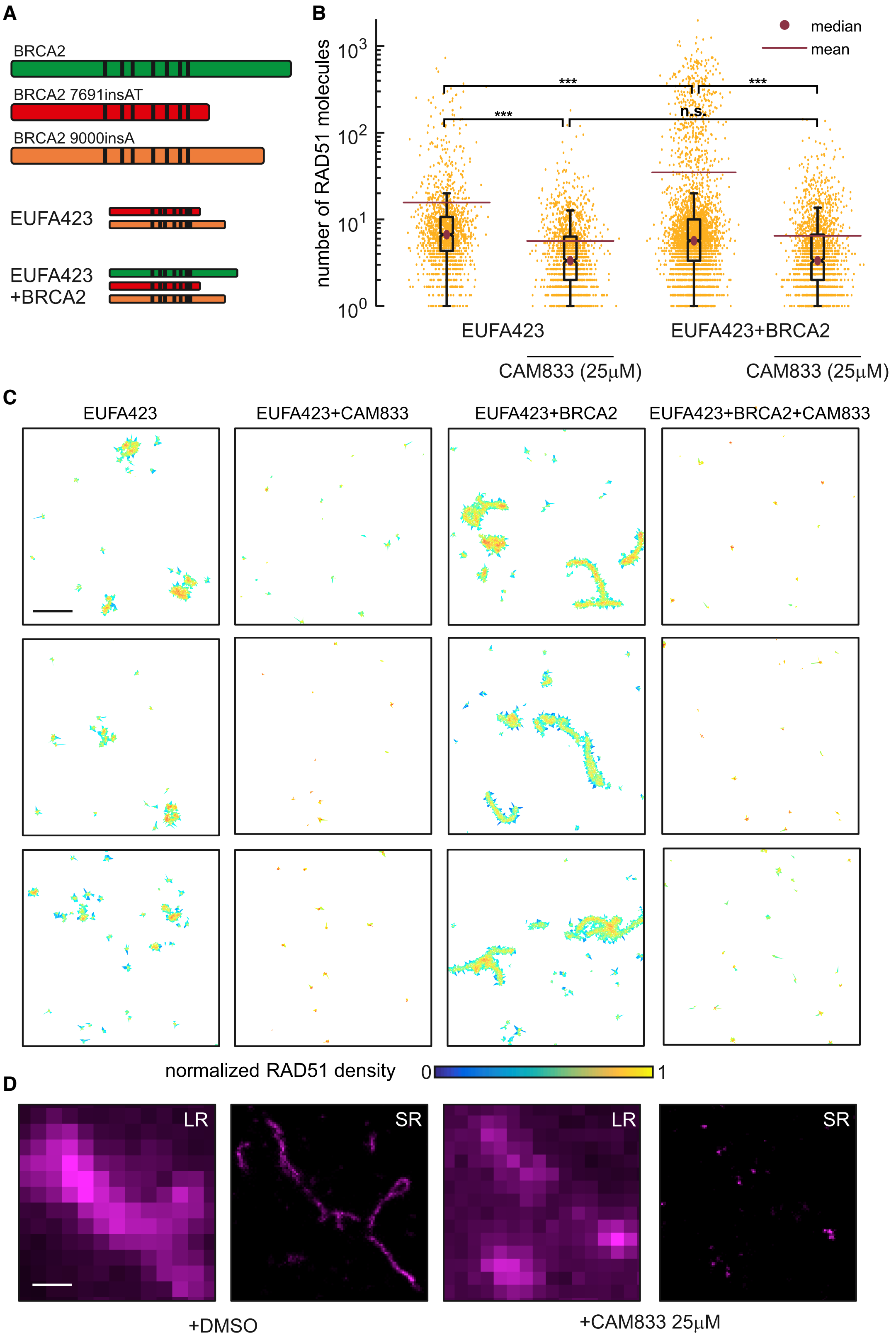

Figure 4. CAM833 inhibits RAD51 molecular clustering at DNA damage sites visualized by SMLM

(A) Diagrammatic representation of the biallelic truncating mutations (red and orange) affecting BRCA2 in the patient-derived cell line EUFA423, and their functional complementation by full-length BRCA2 (green) in EUFA423 + BRCA2 cells. Black vertical lines depict the approximate positions of the BRC repeats.

(legend continued on next page)

The inhibitory effects of **CAM833** are clearly observed by visualization of damage-induced RAD51 clusters as two-dimensional Voronoi polygons scaled to the maximum molecular density (Figure 4C). The compound effectively suppresses RAD51 clustering in both cell types, and in particular, prevents the formation of elongated filaments in control EUFA423 + BRCA2 cells. Example dSTORM pixel images (Figure 4D) further illustrate these effects, providing multiple lines of evidence for CAM833 target engagement and mechanism of action in cells.

CAM833 inhibits DNA repair by homologous recombination

The inhibition of RAD51 clustering at DNA damage sites by **CAM833** prompted us to examine its effect on DNA repair by homologous recombination using the mClover-Lamin A assay (Arnoult et al., 2017; Buisson et al., 2017). In this assay, accurate repair of a nuclease-induced break in a recombination substrate through homologous recombination but not other mechanisms, allows reconstitution of an mClover-Lamin A fluorescent fusion protein, which localizes to the nuclear membrane (Figure 5A). The efficiency of homology-directed repair is readily measured by the enumeration of cells expressing the fusion protein (Figure 5B). We observe (Figure 5C) that **CAM833** induces a dose-dependent decrease in the percentage of cells expressing the fluorescent fusion protein marker. Expression declines in a statistically significant manner at **CAM833** doses between 6.25 and 12.5 μM, to a maximal inhibition at doses above 25 μM. Taken together with its effects on RAD51 clustering (Figure 4), the findings in Figure 5 provide evidence that **CAM833** engages its intracellular target to inhibit DNA repair by homologous recombination.

CAM833 potentiates radiation-induced cell-cycle arrest and increases apoptosis over time

Genetic inactivation of RAD51 enhances cellular sensitivity to ionizing radiation, accompanied by cell-cycle arrest at the G2 checkpoint for DNA damage (Sonoda et al., 1998; Su et al., 2008). We hypothesized that similar effects would be triggered by the exposure of cells to **CAM833**. Indeed, when HCT116 colon carcinoma cells exposed to 20 μM **CAM833** and 3 Gy IR were cell-cycle profiled by flow cytometry 4 to 72 h after exposure, we observed that treatment with **CAM833** causes an increase in the percentage of cells with 4N DNA 4 hours after irradiation. Over time, there is a drop in the percentage of cells with 4N DNA in both treated and control groups. However, whereas in the control the percentage of cells in the apoptotic subG1 fraction remains below 5% throughout, in the compound-treated cells this rises progressively to peak at 15% at 48 hours (Figure 5D). Thus, these results suggest that treatment with **CAM833** increases the progression of G2/M-arrested cells into apoptosis, as opposed to recovery.

CAM833 causes a dose-dependent growth inhibition that is enhanced when combined with ionizing radiation

Consistent with these results, we find that **CAM833** suppresses, in a concentration-dependent manner, the growth of multiple cancer-derived human cell lines (Table S2). For instance, **CAM833** alone inhibits the growth of HCT116 colon carcinoma cells with an average 50% growth inhibition (GI_{50}) value of 38 μM (geometrical mean, n = 18, SD 6.6 μM) after 96 h exposure. Moreover, our results suggest that **CAM833** enhances cellular sensitivity to agents such as IR that induce DNA breakage normally repaired through RAD51-dependent HDR. Thus, when combined with 3 Gy IR, **CAM833** suppresses the growth of HCT116 cells with a GI_{50} of 14 μM (geometrical mean, n = 18, SD, 6.2 μM), a concentration more than 2-fold lower than the GI_{50} for **CAM833** alone (Figure 5E).

These findings prompted us to compare the effects of **CAM833** with those of Carboplatin, a DNA cross-linking agent used in the clinic to sensitize cancers to therapeutic radiation (Clamon et al., 1999). We first exposed cells to a fixed 10 μM dose of either **CAM833** or carboplatin, before treatment with 0 to 3 Gy IR, and compared cell growth using the sulforhodamine B cell proliferation assay 96 h afterwards (Figure 6A). Whereas carboplatin alone is more growth-suppressive than **CAM833** alone, combination with increasing doses of IR potentiates the effects of **CAM833** but not carboplatin (Figure 6A). The concentration-response curves (Figures 6B and 6C) showing the effect of combining 0 to 3 Gy IR with different concentrations of either carboplatin or **CAM833** reflect a complex, dose-dependent response to the combined effects of **CAM833** with IR, leading to changes in the observed IC_{50} (Figure 6D). IR at 1 to 2 Gy sharply potentiates the growth-inhibitory effects of 5×10^{-5} to 5×10^{-4} M doses of **CAM833**. IR at 3 Gy has a smaller effect, across a wider dose range of CAM833. These differences could arise from biological factors such as variations in the amount or type of IR-induced DNA lesions, and/or the relative contribution of HDR to their repair. Collectively, these findings suggest the potential utility of **CAM833** as a radio sensitizer.

CAM833 potentiates PARP1 inhibition in cells wild-type for BRCA2

Cells deficient in RAD51-mediated HDR through the inactivation of tumor suppressor genes like *BRCA1* or *BRCA2* exhibit hypersensitivity to poly-ADP ribose polymerase 1 (PARP1) inhibitors (Bryant et al., 2005; Farmer et al., 2005). We therefore tested whether **CAM833** could potentiate the growth inhibitory effects of PARP1 inhibition by the inhibitor AZD2461 (Jaspers et al., 2013) in cells wild-type for BRCA2 (Figure 7). To this end, we

(B) Distribution of the number of RAD51 molecules contained within damage-induced clusters in EUFA423 or EUFA423 + BRCA2 cells, without or with exposure to 25 μM **CAM833**, 3 h after exposure to 3 Gy ionizing radiation. The box plot was generated using the Matlab boxplot function. The central mark indicates the median (purple dot). The bottom and top edges of the box indicate the 25th and 75th percentiles, respectively. The whiskers extend to the most extreme data points not considered outliers. The mean is marked by a purple line. *** and n.s. indicates p values lower than 10^{-5} and not significant differences, respectively.
(C) Representative SMLM images of RAD51, represented as 2D Voronoi polygons. The color of the polygons shows molecular densities normalized to the maximum value. Scale bar, 500 nm.
(D) High-magnification SMLM images of damage-induced RAD51 filaments in EUFA423 + BRCA2 cells (DMSO-control left-hand panels), and their suppression by **CAM833** (right-hand panels), under the same experimental conditions, at higher magnification. Scale bar, 200 nm. Images are shown either at low-resolution (LR) or super-resolved (SR). Results are representative of two independent repeats.

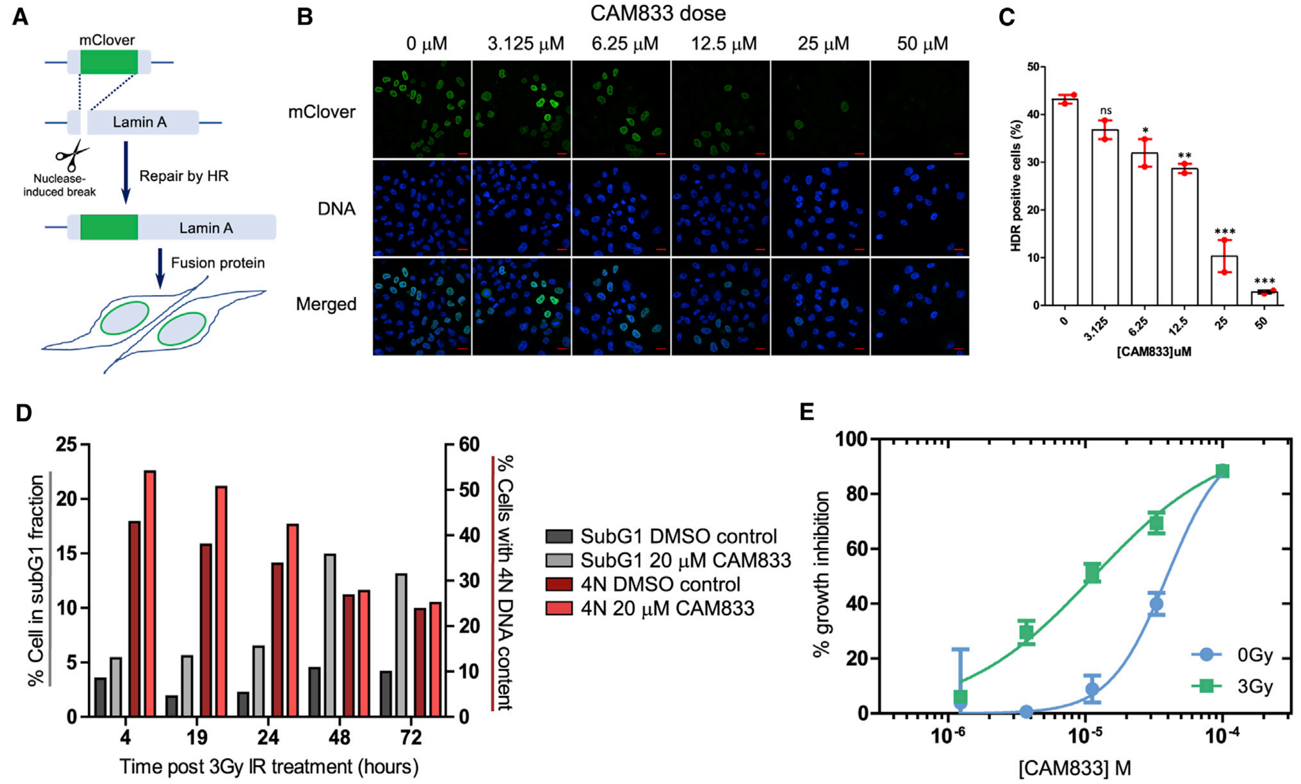

Figure 5. CAM833 inhibits homologous recombination and potentiates cell-cycle arrest

(A) Schematic depiction of the mClover-Lamin assay for DNA repair by homologous DNA recombination (HDR). HeLa Kyoto cells were transfected with the assay plasmids (Lamin A-targeting sgRNA and mClover Lamin A donor constructs) and analyzed for mClover Lamin A-positive (HDR-positive) cells after 3 days. **CAM833** was added to the cells 1 h before transfection and maintained until analysis by microscopy.

(B) Representative microscopic fields showing fluorescence of the mClover-Lamin A fusion protein, DNA (DAPI staining) and merged images after exposure to increasing doses of **CAM833**. Scale bar, 20 μm.

(C) Histogram showing the mean of HDR positive cells (%) ± SE from two independent repeats. More than 200 cells per sample were analyzed in each repeat. Statistical significance was tested by a 1-way ANOVA test, followed by Dunnett's multiple comparison test: ns, p value > 0.05; *p value ≤ 0.05; **p value ≤ 0.01; ***p value ≤ 0.001.

(D) Cell cycle analysis of HCT116 cells over a 72-h time course after treatment with 20 μM **CAM833** or DMSO control, combined with exposure to 3 Gy ionizing radiation.

(E) Dose-response curves for growth inhibition of HCT-116 cells combining 0 (blue circles) or 3 Gy (green squares) of IR at different doses of **CAM833**. Cell growth was measured after 96 h using the sulforhodamine B cell proliferation assay. Each plotted value represents the mean percent growth inhibition ±SEM compared with control cells exposed to DMSO plus the indicated IR dose.

determined dose-response curves for growth inhibition in cells exposed to different doses of AZD2461 combined with a fixed dose of either 10 μM (Figure 7A) or 20 μM (Figure 7B) of **CAM833**. While **CAM833** alone had little effect (blue triangles), its combination with AZD2461 potentiated the growth-suppressive effects of PARP1 inhibition in a dose-dependent manner. Reciprocally, we also measured the dose-response curves for growth inhibition in cells exposed to different doses of **CAM833** combined with a fixed dose of either 0.1 μM (Figure 7C) or 1 μM (Figure 7D) of AZD2461. These doses of AZD2461 have little effect when administered alone (blue diamonds), but again, their combination with **CAM833** potentiates growth suppression by PARP1 inhibition in cells wild-type for BRCA2.

DISCUSSION

We report here the discovery of CAM833, a sub-micromolar chemical inhibitor of the regulatory protein-protein interaction between the RAD51 recombinase and the BRC repeat motifs of the tumor suppressor BRCA2. Using structure determination by X-ray crystallography, we show that **CAM833** engages with two hydrophobic pockets on the surface of RAD51 that normally accommodate conserved hydrophobic side chains from the BRC repeats of BRCA2, thereby directly competing with the RAD51:BRCA2 interaction. These pockets also normally mediate RAD51 multimerization on DNA substrates during the process that leads to HDR, by accommodating corresponding hydrophobic residues from an adjacent RAD51 protomer to form the protomer-protomer interface. Consistent with these structural considerations, we show that CAM833 suppresses the assembly of RAD51 into damage-induced filaments visualized by single-molecule localization microscopy, and impairs DNA repair by homologous DNA recombination. Moreover, we present multiple lines of evidence suggesting that **CAM833** potentiates growth inhibition, cell-cycle arrest and cytotoxicity induced by DNA damage, consistent with its predicted ability

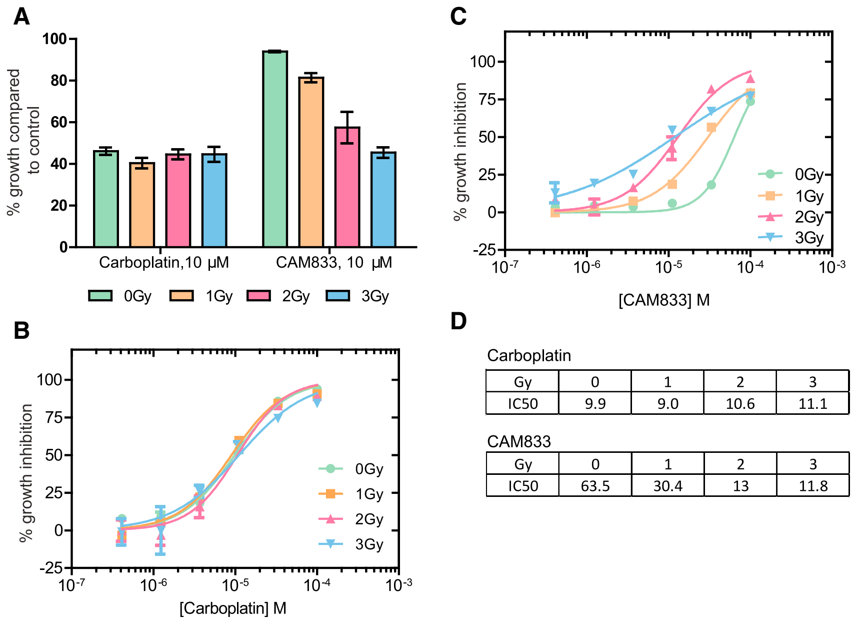

Carboplatin

Gy	0	1	2	3
IC50	9.9	9.0	10.6	11.1

CAM833

Gy	0	1	2	3
IC50	63.5	30.4	13	11.8

Figure 6. Low-dose ionizing radiation potentiates the effects of CAM833 but not carboplatin

(A) Cell growth after exposure to increasing levels of ionizing radiation in the presence of a fixed dose (10 μM) of either Carboplatin or **CAM833**. Bars depict percent growth compared with control cells exposed to DMSO plus the indicated IR dose, represented as the mean ± SEM. Values <100 indicate growth inhibition.

(B and C) Plot dose-response curves for growth inhibition combining 0 (green circles), 1 Gy (orange squares), 2 Gy (red triangles), or 3 Gy (blue triangles) of IR with different doses of carboplatin (B) or **CAM833** (C) shown as molar. In (B) and (C), each plotted value represents the mean percent growth inhibition ±SEM compared with control cells exposed to DMSO plus the indicated IR dose.

(D) Observed changes in IC_{50} (expressed in μM) for growth inhibition derived from the curves in (B) and (C). These data are representative of 3 independent experiments.

to suppress DNA repair by HDR. Our findings have several important implications.

CAM833 is a well-characterized, selective chemical probe molecule that should prove valuable for further elucidating the biology of the RAD51-BRCA2 protein-protein interaction and the associated HDR pathways. Moreover, **CAM833** is a chemically tractable starting point for the further, structure-guided development of optimized inhibitory compounds with the potential for development into a drug compound suitable for clinical studies. The development of this molecule through an innovative strategy of combining a fragment hit with a peptide lead compound reveals what is likely to be a generally applicable strategy for the development of inhibitors of protein-protein interactions featuring a continuous peptide epitope (Scott et al., 2016).

Our work exemplifies a strategy to modulate the activity of RAD51 during HDR through two of its key regulatory protein-protein interactions. The first of these interactions is between RAD51 and the BRC repeats of BRCA2, which is essential to target RAD51 to cellular sites of DNA damage, and may also regulate RAD51 assembly on DNA substrates at these sites. The second interaction blocked by CAM833 is between RAD51 protomers, which occurs at the same structural motif engaged by the BRC repeats, and enables RAD51 assembly by multimerization. Our findings provide several lines of evidence that

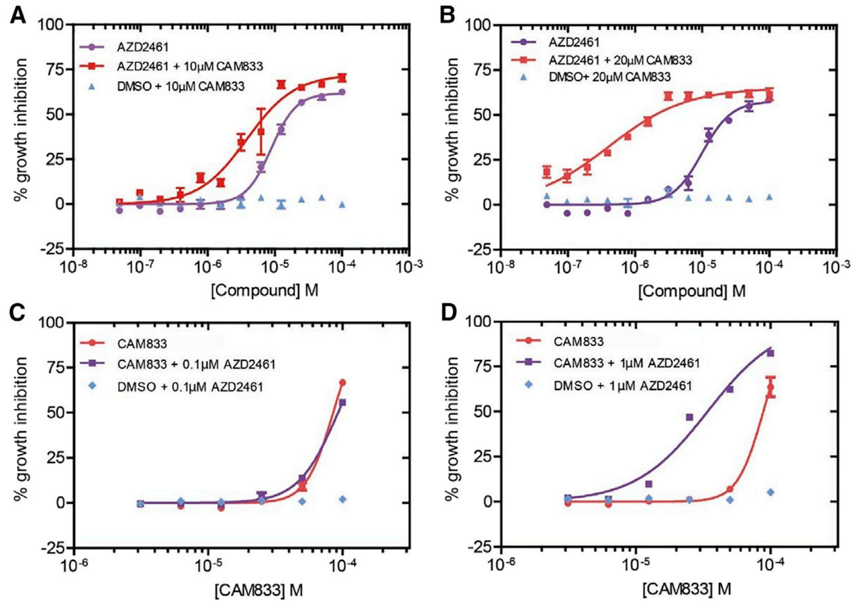

Figure 7. CAM833 potentiates the growth suppressive effect of PARP1 inhibition in BRCA2 wild-type cells

(A and B) Dose-response curves for growth inhibition in HCT116 cells exposed to different doses of AZD2461 plotted as molar combined with a fixed dose of either 10 μM (A) or 20 μM (B) of **CAM833**. Control experiments in which vehicle (DMSO) was added in place of AZD2461 are plotted in blue. Growth was measured 96 h after compound exposure using the SRB assay, and is depicted as the mean percent inhibition ±SEM compared with controls.

(C and D) Reciprocal dose-response curves for growth inhibition after exposure to different doses of **CAM833** plotted as molar combined with a fixed dose of either 0.1 μM (C) or 1 μM (D) of AZD2461. Control experiments in which vehicle (DMSO) was added in place of **CAM833** are plotted in blue. Measurements and plots are as in the previous panels. Results are representative of two independent repeats.

CAM833 acts in cells to engage RAD51 and block the protein-protein interactions that lead to its multimerization at sites of DNA damage. We find using SMLM by d-STORM that **CAM833** suppresses the molecular clustering of RAD51 at damage sites, and prevents the extension of these clusters into extended RAD51 filaments, providing evidence for target engagement and the proposed mechanism of action. The mechanism of **CAM833** action via the inhibition of RAD51-mediated HDR is further supported by our finding that the compound sensitizes cells with wild-type BRCA2 to the growth inhibitory effects of the PARP1 inhibitor, AZD2461. In the context of wild-type BRCA2, PARP1 inhibition alone is usually ineffective. While these results further support the cellular mechanism underlying **CAM833** action, we are skeptical that systemic inhibition of RAD51 combined with the systemic effects of PARP1 inhibition has therapeutic potential owing to the likelihood of dose-limiting mechanism-related toxicity in normal tissues.

However, **CAM833** also potentiates the cellular effects of ionizing radiation, a potent inducer of DNA breakage. When combined with IR, CAM833 sensitizes cells to IR-induced cell-cycle arrest at the G2/M phase of the cell cycle, and enhances cell death by apoptosis. Collectively, these findings provide evidence supporting the further development of small-molecule inhibitors of the regulatory protein-protein interactions of RAD51 for cancer therapy through radiosensitisation. Indeed, the locoregional nature of radiation therapy may prove in this regard to have a higher therapeutic index.

SIGNIFICANCE

Protein-protein interactions that mediate intracellular reactions leading to the repair of damaged DNA are an important target for anti-cancer drug discovery. Here, we report using structure-guided lead discovery the development of a potent orthosteric inhibitor, CAM833, of the protein-protein interaction between the BRCA2 tumor suppressor and the RAD51 recombinase, which is critical for the error-free repair of DNA breakage by homologous DNA recombination. The significance of our work is 3-fold. First, it exemplifies a strategy for the development of inhibitors that target protein-protein interactions wherein a contiguous series of amino acids interact with a protein surface, by merging a peptidic inhibitor derived from those amino acids with chemical fragment hits identified by biophysical and crystallographic screening. Second, we demonstrate using single-molecule localization ("super-resolution") microscopy that CAM833 inhibits RAD51 molecular clustering to prevent the assembly of extended RAD51 filaments at sites of DNA damage, validating target engagement, and demonstrating a unique mechanism of action. Finally, we show that CAM833 inhibits the cellular response to DNA damage, potentiating in BRCA2 wild-type cells the cytotoxic effects both of ionizing radiation or of PARP1 inhibitors, opening future avenues for anti-cancer drug development.

STAR★METHODS

Detailed methods are provided in the online version of this paper and include the following:

- KEY RESOURCES TABLE
- RESOURCE AVAILABILITY
  - Lead contact
  - Materials availability
  - Data and code availability
- EXPERIMENTAL MODEL AND SUBJECT DETAILS
  - *E. coli* for protein production
  - Cell line and cell culture
- METHOD DETAILS
  - Chemical synthesis
  - Protein production
  - ITC
  - FP assay
  - X-ray crystallography
  - Immunofluorescent visualisation of RAD51 foci/γH2AX foci in A549 cells using the Cellomics Arrayscan V^{ti} high content microscopy
  - SRB growth inhibition assay
  - Flow cytometry
  - Super-resolution microscopy
  - Cluster data analysis
  - mClover Lamin A HDR assay
- QUANTIFICATION AND STATISTICAL ANALYSES

SUPPLEMENTAL INFORMATION

Supplemental Information can be found online at https://doi.org/10.1016/j.chembiol.2021.02.006.

ACKNOWLEDGMENTS:

We thank Dr. Adrian Schreyer for developing a database for data management and Dr. Tara Pukala and Prof. Carol Robinson for taking part in the early stages of this project. We are grateful for Diamond Light Source for access to beamlines I02, I04 and I24 (proposals mx315 and mx7141) and ESRF for access beamline ID14-4. We thank the X-ray crystallographic and biophysics facilities at the Department of Biochemistry for support and access. This work was funded by the Wellcome Trust Translational Award (080083/Z/06/Z) and Seeding Drug Discovery Award (091058/Z/09/Z) and we acknowledge the support of their Seeding Drug Discovery team, in particularly Dr. Sarah Hardy and Prof. Chas Bountra, for useful discussions. This work was also funded by Medical Research Council (MRC) Program grants MC_UU_12022/1 and MC_UU_12022/8 to ARV, and a research grant from Astex Pharmaceuticals. We thank WuXi AppTec for Pharmacokinetic and cell-line sensitivity data and Cyprotex for ADMET data. C.A., senior author of this paper and a good friend and colleague of the remaining authors, sadly died while this manuscript was in revision; we dedicate this paper to him.

AUTHOR CONTRIBUTIONS

Experimental investigations in Figures 1 and 2 were carried out by D.E.S., T.P.S., A.G.C., C.D., G.F., C.V., T.M., A.R.B., M.E.M., R.S., D.H., A.H., M.E., T.P., and J.S.; in Figures 3, 5, 6, and 7, by N.J.F.-N. and L.B.; in Figure 4, by K.T.H. and A.E.; and in Figure 5 by M.L., A.E., and B.H. T.L.B., A.R.V., C.A., M.H., L.P., G.M., J.S., and T.P. conceptualized the project, supervised the experimental work, and analyzed the results. D.E.S., N.J.F.-N., J.S., M.H., C.A., and A.R.V. wrote the manuscript with input from all the authors. Each corresponding author supervised and is responsible for distinct aspects of this multidisciplinary project. Chemistry was led by J.S. and C.A.; biochemistry and structural biology by M.H.; and microscopy, cell genetics, and cell biology by A.R.V.

DECLARATION OF INTERESTS

Venkitaraman, Pellegrini, Blundell et al., WO2004035621 - Use of crystal structure of human RAD51-BRCA2 repeat complex in screening for anti tumor agents.

Received: July 16, 2020
Revised: December 18, 2020
Accepted: February 3, 2021
Published: March 3, 2021

REFERENCES

Adams, P.D., Afonine, P.V., Bunkóczi, G., Chen, V.B., Davis, I.W., Echols, N., Headd, J.J., Hung, L.-W., Kapral, G.J., Grosse-Kunstleve, R.W., et al. (2010). PHENIX: a comprehensive Python-based system for macromolecular structure solution. Acta Crystallogr. D. Biol. Crystallogr. *66*, 213–221.

Arnoult, N., Correia, A., Ma, J., Merlo, A., Garcia-Gomez, S., Maric, M., Tognetti, M., Benner, C.W., Boulton, S.J., Saghatelian, A., et al. (2017). Regulation of DNA repair pathway choice in S and G2 phases by the NHEJ inhibitor CYREN. Nature *549*, 548–552.

Bagnolini, G., Milano, D., Manerba, M., Schipani, F., Ortega, J.A., Gioia, D., Falchi, F., Balboni, A., Farabegoli, F., De Franco, F., et al. (2020). Synthetic lethality in pancreatic cancer: discovery of a new RAD51-BRCA2 small molecule disruptor that inhibits homologous recombination and synergizes with olaparib. J. Med. Chem. *63*, 2588–2619.

Bignell, G., Micklem, G., Stratton, M.R., Ashworth, A., and Wooster, R. (1997). The BRC repeats are conserved in mammalian BRCA2 proteins. Hum. Mol. Genet. *6*, 53–58.

Blundell, T.L., Jhoti, H., and Abell, C. (2002). High-throughput crystallography for lead discovery in drug design. Nat. Rev. Drug Discov. *1*, 45–54.

Brouwer, I., Moschetti, T., Candelli, A., Garcin, E.B., Modesti, M., Pellegrini, L., Wuite, G.J., and Peterman, E.J. (2018). Two distinct conformational states define the interaction of human RAD51- ATP with single-stranded DNA. EMBO J. *37*, e98162.

Bryant, H.E., Schultz, N., Thomas, H.D., Parker, K.M., Flower, D., Lopez, E., Kyle, S., Meuth, M., Curtin, N.J., and Helleday, T. (2005). Specific killing of BRCA2-deficient tumours with inhibitors of poly(ADP-ribose) polymerase. Nature *434*, 913–917.

Budke, B., Kalin, J.H., Pawlowski, M., Zelivianskaia, A.S., Wu, M., Kozikowski, A.P., and Connell, P.P. (2012a). An optimized RAD51 inhibitor that disrupts homologous recombination without requiring Michael acceptor reactivity. J. Med. Chem. *56*, 254–263.

Budke, B., Logan, H.L., Kalin, J.H., Zelivianskaia, A.S., Cameron McGuire, W., Miller, L.L., Stark, J.M., Kozikowski, A.P., Bishop, D.K., and Connell, P.P. (2012b). RI-1: a chemical inhibitor of RAD51 that disrupts homologous recombination in human cells. Nucleic Acids Res. *40*, 7347–7357.

Budke, B., Tueckmantel, W., Miles, K., Kozikowski, A.P., and Connell, P.P. (2019). Optimization of drug candidates that inhibit the D-loop activity of RAD51. ChemMedChem *14*, 1031–1040.

Buisson, R., Niraj, J., Rodrigue, A., Kwen Ho, C., Krauzer, J., Foo, T.K., Hardy, E.L.-J., Dellaire, G., Hass, W., Xia, B., et al. (2017). Coupling of homologous recombination and the checkpoint by ATR. Mol. Cell *65*, 336–346.

Carreira, A., and Kowalczykowski, S.C. (2011). Two classes of BRC repeats in BRCA2 promote RAD51 nucleoprotein filament function by distinct mechanisms. Proc. Natl. Acad. Sci. U S A *108*, 10448–10453.

Carreira, A., Hilario, J., Amitani, I., Baskin, R.J., Shivji, M.K.K., Venkitaraman, A.R., and Kowalczykowski, S.C. (2009). The BRC repeats of BRCA2 modulate the DNA-binding selectivity of RAD51. Cell *136*, 1032–1043.

Chen, C.-F., Chen, P.-L., Zhong, Q., Sharp, Z.D., and Lee, W.-H. (1999a). Expression of BRC repeats in breast cancer cells disrupts the BRCA2-Rad51 complex and leads to radiation hypersensitivity and loss of G 2/M checkpoint control. J. Biol. Chem. *274*, 32931–32935.

Chen, G., Yuan, S.S.F., Liu, W., Xu, Y., Trujillo, K., Song, B., Cong, F., Goff, S.P., Wu, Y., Arlinghaus, R., et al. (1999b). Radiation-induced assembly of Rad51 and Rad52 recombination complex requires ATM and c-Abl. J. Biol. Chem. *274*, 12748–12752.

Clamon, G., Herndon, J., Cooper, R., Chang, A.Y., Rosenman, J., and Green, M.R. (1999). Radiosensitization with carboplatin for patients with unresectable stage III non–small-cell lung cancer: a phase III trial of the Cancer and Leukemia Group B and the Eastern Cooperative Oncology Group. J. Clin. Oncol. *17*, 4.

Cole, D.J., Janecek, M., Stokes, J.E., Rossmann, M., Faver, J.C., McKenzie, G.J., Venkitaraman, A.R., Hyvönen, M., Spring, D.R., Huggins, D.J., et al. (2017). Computationally-guided optimization of small-molecule inhibitors of the Aurora A kinase–TPX2 protein–protein interaction. Chem. Commun. *53*, 9372–9375.

Conway, A.B., Lynch, T.W., Zhang, Y., Fortin, G.S., Fung, C.W., Symington, L.S., and Rice, P.A. (2004). Crystal structure of a Rad51 filament. Nat. Struct. Mol. Biol. *11*, 791–796.

Coyne, A.G., Scott, D.E., and Abell, C. (2010). Drugging challenging targets using fragment-based approaches. Curr. Opin. Chem. Biol. *14*, 299–307.

Davies, O.R., and Pellegrini, L. (2007). Interaction with the BRCA2 C terminus protects RAD51–DNA filaments from disassembly by BRC repeats. Nat. Struct. Mol. Biol. *14*, 475–483.

Davies, A.A., Masson, J.Y., McIlwraith, M.J., Stasiak, A.Z., Stasiak, A., Venkitaraman, A.R., and West, S.C. (2001). Role of BRCA2 in control of the RAD51 recombination and DNA repair protein. Mol. Cell *7*, 273–282.

Esashi, F., Galkin, V.E., Yu, X., Egelman, E.H., and West, S.C. (2007). Stabilization of RAD51 nucleoprotein filaments by the C-terminal region of BRCA2. Nat. Struct. Mol. Biol. *14*, 468–474.

Falchi, F., Giacomini, E., Masini, T., Boutard, N., Di Ianni, L., Manerba, M., Farabegoli, F., Rossini, L., Robertson, J., Minucci, S., et al. (2017). Synthetic lethality triggered by combining olaparib with BRCA2–Rad51 disruptors. ACS Chem. Biol. *12*, 2491–2497.

Farmer, H., McCabe, N., Lord, C.J., Tutt, A.N.J., Johnson, D.A., Richardson, T.B., Santarosa, M., Dillon, K.J., Hickson, I., Knights, C., et al. (2005). Targeting the DNA repair defect in BRCA mutant cells as a therapeutic strategy. Nature *434*, 917–921.

Haas, K.T., Lee, M., Esposito, A., and Venkitaraman, A.R. (2018). Single-molecule localization microscopy reveals molecular transactions during RAD51 filament assembly at cellular DNA damage sites. Nucleic Acids Res. *46*, 2398–2416.

Hattori, H., Skoulidis, F., Russell, P., and Venkitaraman, A.R. (2011). Context dependence of checkpoint kinase 1 as a therapeutic target for pancreatic cancers deficient in the BRCA2 tumor suppressor. Mol. Cancer Ther. *10*, 670–678.

Howlett, N.G., Taniguchi, T., Olson, S., Cox, B., Waisfisz, Q., De Die-Smulders, C., Persky, N., Grompe, M., Joenje, H., Pals, G., et al. (2002). Biallelic inactivation of BRCA2 in Fanconi anemia. Science *297*, 606–609.

Huang, F., and Mazin, A.V. (2014). A small molecule inhibitor of human RAD51 potentiates breast cancer cell killing by therapeutic agents in mouse xenografts. PLoS One *9*, e100993.

Huang, F., Motlekar, N.A., Burgwin, C.M., Napper, A.D., Diamond, S.L., and Mazin, A.V. (2011). Identification of specific inhibitors of human RAD51 recombinase using high-throughput screening. ACS Chem. Biol. *6*, 628–635.

Ishida, T., Takizawa, Y., Kainuma, T., Inoue, J., Mikawa, T., Shibata, T., Suzuki, H., Tashiro, S., and Kurumizaka, H. (2009). DIDS, a chemical compound that inhibits RAD51-mediated homologous pairing and strand exchange. Nucleic Acids Res. *37*, 3367–3376.

Jaspers, J.E., Kersbergen, A., Boon, U., Sol, W., van Deemter, L., Zander, S.A., Drost, R., Wientjens, E., Ji, J., Aly, A., et al. (2013). Loss of 53BP1 causes PARP inhibitor resistance in Brca1 -mutated mouse mammary tumors. Cancer Discov. *3*, 68–81.

Jeyasekharan, A.D., Liu, Y., Hattori, H., Pisupati, V., Jonsdottir, A.B., Rajendra, E., Lee, M., Sundaramoorthy, E., Schlachter, S., Kaminski, C.F., et al. (2013). A cancer-associated BRCA2 mutation reveals masked nuclear export signals controlling localization. Nat. Struct. Mol. Biol. *20*, 1191–1198.

Kabsch, W. (2010). XDS. Acta Crystallogr. D Biol. Crystallogr. *66*, 125–132.

CellPress
OPEN ACCESS

Lv, W., Budke, B., Pawlowski, M., Connell, P.P., and Kozikowski, A.P. (2016). Development of small molecules that specifically inhibit the D-loop activity of RAD51. J. Med. Chem. *59*, 4511–4525.

Moschetti, T., Sharpe, T., Fischer, G., Marsh, M.E., Ng, H.K., Morgan, M., Scott, D.E., Blundell, T.L., R Venkitaraman, A., Skidmore, J., et al. (2016). Engineering archeal surrogate systems for the development of protein–protein interaction inhibitors against human RAD51. J. Mol. Biol. *428*, 4589–4607.

Nomme, J., Renodon-Cornière, A., Asanomi, Y., Sakaguchi, K., Stasiak, A.Z., Stasiak, A., Norden, B., Tran, V., and Takahashi, M. (2010). Design of potent inhibitors of human RAD51 recombinase based on BRC motifs of BRCA2 protein: modeling and experimental validation of a chimera peptide. J. Med. Chem. *53*, 5782–5791.

Normand, A., Rivière, E., and Renodon-Cornière, A. (2014). Identification and characterization of human Rad51 inhibitors by screening of an existing drug library. Biochem. Pharmacol. *91*, 293–300.

Pastushok, L., Fu, Y., Lin, L., Luo, Y., DeCoteau, J.F., Lee, K., and Geyer, C.R. (2019). A novel cell-penetrating antibody fragment inhibits the DNA repair protein RAD51. Sci. Rep. *9*, 11227.

Pellegrini, L., Yu, D.S., Lo, T., Anand, S., Lee, M., Blundell, T.L., and Venkitaraman, A.R. (2002). Insights into DNA recombination from the structure of a RAD51-BRCA2 complex. Nature *420*, 287–293.

Rajendra, E., and Venkitaraman, A.R. (2010). Two modules in the BRC repeats of BRCA2 mediate structural and functional interactions with the RAD51 recombinase. Nucleic Acids Res. *38*, 82–96.

Roberti, M., Schipani, F., Bagnolini, G., Milano, D., Giacomini, E., Falchi, F., Balboni, A., Manerba, M., Farabegoli, F., De Franco, F., et al. (2019). Rad51/BRCA2 disruptors inhibit homologous recombination and synergize with olaparib in pancreatic cancer cells. Eur. J. Med. Chem. *165*, 80–92.

Rogakou, E.P., Pilch, D.R., Orr, A.H., Ivanova, V.S., and Bonner, W.M. (1998). DNA double-stranded breaks induce histone H2AX phosphorylation on serine 139. J. Biol. Chem. *273*, 5858–5868.

Schneider, C., Rasband, W., and Eliceiri, K. (2012). NIH Image to ImageJ: 25 years of image analysis. Nat Methods *9*, 671–675.

Scott, D.E., Ehebauer, M.T., Pukala, T., Marsh, M., Blundell, T.L., Venkitaraman, A.R., Abell, C., and Hyvönen, M. (2013). Using a fragment-based approach to target protein-protein interactions. Chembiochem *14*, 332–342.

Scott, D.E., Marsh, M., Blundell, T.L., Abell, C., and Hyvönen, M. (2016). Structure-activity relationship of the peptide binding-motif mediating the BRCA2:RAD51 protein-protein interaction. FEBS Lett. *590*, 1094–1102.

Shin, D.S., Pellegrini, L., Daniels, D.S., Yelent, B., Craig, L., Bates, D., Yu, D.S., Shivji, M.K., Hitomi, C., Arvai, A.S., et al. (2003). Full-length archaeal Rad51 structure and mutants: mechanisms for RAD51 assembly and control by BRCA2. EMBO J. *22*, 4566–4576.

Shivji, M.K.K., Mukund, S.R., Rajendra, E., Chen, S., Short, J.M., Savill, J., Klenerman, D., and Venkitaraman, A.R. (2009). The BRC repeats of human BRCA2 differentially regulate RAD51 binding on single- versus double-stranded DNA to stimulate strand exchange. Proc. Natl. Acad. Sci. U S A *106*, 13254–13259.

Short, J.M., Liu, Y., Chen, S., Soni, N., Madhusudhan, M.S., Shivji, M.K.K., and Venkitaraman, A.R. (2016). High-resolution structure of the presynaptic RAD51 filament on single-stranded DNA by electron cryo-microscopy. Nucleic Acids Res. *44*, 9017–9030.

Sonoda, E., Sasaki, M.S., Buerstedde, J.M., Bezzubova, O., Shinohara, A., Ogawa, H., Takata, M., Yamaguchi-Iwai, Y., and Takeda, S. (1998). Rad51-deficient vertebrate cells accumulate chromosomal breaks prior to cell death. EMBO J. *17*, 598–608.

Su, X., Bernal, J.A., and Venkitaraman, A.R. (2008). Cell-cycle coordination between DNA replication and recombination revealed by a vertebrate N-end rule degron-Rad51. Nat. Struct. Mol. Biol. *15*, 1049–1058.

Takaku, M., Kainuma, T., Ishida-Takaku, T., Ishigami, S., Suzuki, H., Tashiro, S., van Soest, R.W.M., Nakao, Y., and Kurumizaka, H. (2011). Halenaquinone, a chemical compound that specifically inhibits the secondary DNA binding of RAD51. Genes Cells *16*, 427–436.

Trenner, A., Godau, J., and Sartori, A.A. (2018). A short BRCA2-derived cell-penetrating peptide targets RAD51 function and confers hypersensitivity toward PARP inhibition. Mol. Cancer Ther. *17*, 1392–1405.

Turchick, A., Hegan, D.C., Jensen, R.B., and Glazer, P.M. (2017). A cell-penetrating antibody inhibits human RAD51 via direct binding. Nucleic Acids Res. *45*, 11782–11799.

Turchick, A., Liu, Y., Zhao, W., Cohen, I., and Glazer, P.M. (2019). Synthetic lethality of a cell-penetrating anti-RAD51 antibody in PTEN-deficient melanoma and glioma cells. Oncotarget *10*, 1272–1283.

Venkitaraman, A.R. (2014). Tumour suppressor mechanisms in the control of chromosome stability: insights from BRCA2. Mol. Cells *37*, 95–99.

Vonrhein, C., Flensburg, C., Keller, P., Sharff, A., Smart, O., Paciorek, W., Womack, T., and Bricogne, G. (2011). Data processing and analysis with the autoPROC toolbox. Acta Crystallogr. D Biol. Crystallogr. *67*, 293–302.

Vydyam, P., Dutta, D., Sutram, N., Bhattacharyya, S., and Bhattacharyya, M.K. (2019). A small-molecule inhibitor of the DNA recombinase Rad51 from Plasmodium falciparum synergizes with the antimalarial drugs artemisinin and chloroquine. J. Biol. Chem. *294*, 8171–8183.

Wang, Z.X. (1995). An exact mathematical expression for describing competitive binding of two different ligands to a protein molecule. FEBS Lett. *360*, 111–114.

Ward, A., Dong, L., Harris, J.M., Khanna, K.K., Al-Ejeh, F., Fairlie, D.P., Wiegmans, A.P., and Liu, L. (2017). Quinazolinone derivatives as inhibitors of homologous recombinase RAD51. Bioorg. Med. Chem. Lett. *27*, 3096–3100.

West, S.C. (2003). Molecular views of recombination proteins and their control. Nat. Rev. Mol. Cell Biol. *4*, 435–445.

Wong, A.K.C., Pero, R., Ormonde, P.A., Tavtigian, S.V., and Bartel, P.L. (1997). RAD51 interacts with the evolutionarily conserved BRC motifs in the human breast cancer susceptibility gene brca2. J. Biol. Chem. *272*, 31941–31944.

Xu, J., Zhao, L., Xu, Y., Zhao, W., Sung, P., and Wang, H. (2017). Cryo-EM structures of human RAD51 recombinase filaments during catalysis of DNA-strand exchange. Nat. Struct. Mol. Biol. *24*, 40–46.

Zhu, J., Zhou, L., Wu, G., Konig, H., Lin, X., Li, G., Qiu, X.-L., Chen, C.-F., Hu, C.-M., Goldblatt, E., et al. (2013). A novel small molecule RAD51 inactivator overcomes imatinib-resistance in chronic myeloid leukaemia. EMBO Mol. Med. *5*, 353–365.

Zhu, J., Chen, H., Guo, X.E., Qiu, X., Hu, C., Chamberlin, A.R., and Lee, W. (2015). Synthesis, molecular modeling, and biological evaluation of novel RAD51 inhibitors. Eur. J. Med. Chem. *96*, 196–208.

STAR★METHODS

KEY RESOURCES TABLE

REAGENT or RESOURCE	SOURCE	IDENTIFIER
Antibodies		
Mouse polyclonal anti-RAD51 Antibody	Abnova	Cat# H00005888-B01P; RRID: AB_1522243
Alexa Fluor 488 labelled anti-mouse secondary antibody	Invitrogen	Cat# A11001; RRID: AB_2534069
Anti-phospho γH2AX primary mouse monoclonal antibody	Millipore	Cat#05-636; RRID: AB_309864
Rabbit anti-RAD51 monoclonal antibody	Abcam	Cat# ab 133534; RRID: AB_2722613
Bacterial and virus strains		
BL21(DE3) *E. coli* strain	New England Biolabs	C2527I
Chemicals, Peptides, and Recombinant Proteins		
ChimRAD51 protein	Moschetti et al., 2016	N/A
Alexa Fluor 488-labelled BRC4 peptide	Moschetti et al., 2016	N/A
HumRadA1 protein	Moschetti et al., 2016	N/A
HumRadA22F protein	Moschetti et al., 2016	N/A
RAD51 protein	Moschetti et al., 2016	N/A
Tetrapeptide (**1**)	Scott et al., 2016	FHTA
Inhibitors 4-6 and CAM833	Synthesis from commercially available materials described in this paper	CAM833
AZD2461	SelleckChem	Cat#S7029
MEA-HCl	Sigma	M6500
Glucose oxidase	Sigma	G2133
Catalase	Sigma	C100
Deposited data		
Crystallographic coordinates of Rad51 in complex with BRC4 repeat	Pellegrini et al., 2002	PDB: 1N0W
HumRadA2 in complex with oligomerization peptide	Moschetti et al., 2016	PDB: 5NWL
HumRadA1 in complex with 2-naphthol	Scott et al., 2013	PDB: 4B32
HumRadA1 in complex with FHTA tetrapeptide	Scott et al., 2013	PDB: 4B3B
Crystallographic coordinates and structure factors for **3** in complex with HumRadA1	This paper	PDB: 6TV3
Crystallographic coordinates and structure factors for **4** in complex with HumRadA1	This paper	PDB: 6TWR
Crystallographic coordinates and structure factors for **6** in complex with HumRadA22F	This paper	PDB: 6TW4
Crystallographic coordinates and structure factors for **6** in complex with HumRadA33F	This paper	PDB: 6XTW
Crystallographic coordinates and structure factors for **CAM833** in complex with HumRadA22F	This paper	PDB: 6TW9
Experimental models: cell lines		
HCT116 colon carcinoma cells	ATCC	CCL-185
A549 lung adenocarcinoma cells	ATCC	CCL-247
HeLa Kyoto cells	From the laboratory of Jonathan Pines, Institute of Cancer Research, London	N/A

(*Continued on next page*)

Continued

REAGENT or RESOURCE	SOURCE	IDENTIFIER
EUFA423 cells	From the European Fanconi Anemia Registry, VU University Medical Center, Amsterdam, The Netherlands	N/A
EUFA423+BRCA2	Hattori et al., 2011	N/A
Recombinant DNA		
pUC CBA-SpCas9.EF1a-BFP.sgLMNA	Arnoult et al., 2017	Addgene Plasmid #98971
pCAGGS Donor mClover-LMNA	Arnoult et al., 2017	Addgene Plasmid #98970
Plasmids for expression of HumRadA and ChimRAD51 proteins and BRC4 repeat	Moschetti et al., 2016	N/A
Software and algorithms		
Origin for ITC200	Malvern Instruments	https://www.malvernpanalytical.com/en
XDS	MPI for Medical Research	http://xds.mpimf-heidelberg.mpg.de/
Autoproc	Global Phasing	https://www.globalphasing.com/autoproc/
Phenix.refine	Phenix consortium	https://www.phenix-online.org/
autoBuster	Global Phasing	https://www.globalphasing.com/buster/
Prism	GraphPad	https://www.graphpad.com/scientific-software/prism/
FCS Express	De Novo software	https://denovosoftware.com/
Matlab with Grafeo plugin	MathWorks	https://www.mathworks.com/products/matlab.html, https://github.com/inatamara/Grafeo-dSTORM-analysis
Chemdraw 16	PerkinElmer	https://www.perkinelmer.com/category/chemdraw
Pro Fit	Quan Soft	https://www.quansoft.com
ImageJ	Schneider et al., 2012	https://imagej.nih.gov/ij/

RESOURCE AVAILABILITY

Lead contact

Further information and requests for resources and reagents should be directed to and will be fulfilled by the Lead Contact, John Skidmore (js930@cam.ac.uk).

Materials availability

Plasmids for the production of RAD51 surrogate protein and BRC4 peptide are available from MH on request. Synthetic routes to all chemical compounds are described using established methodology from commercially available compounds. Where available these may be shared by the lead contact. Antibodies, reagents and cell lines used for the biological studies were obtained from the commercial or academic sources described in the attached Key resources table.

Data and code availability

All crystallographic statistics are shown in Table S3 and coordinates and structure factors deposited in the Protein Data Bank under accession numbers 6TV3, 6TWR, 6TW4 6XTW and 6TW9.

Software used for the biological studies were obtained from the commercial or academic sources described in the STAR methods Key resources table.

EXPERIMENTAL MODEL AND SUBJECT DETAILS

E. coli for protein production

For recombinant protein production the BL21(DE3) strain of E. coli (New England Biolabs, USA) was used in the Hyvönen Lab. Cells were grown in 2YT medium at 37°C until OD_{600} of 0.8-1.0 after which the expression was induced with 400 μM IPTG. Cells were grown for further 3 hours, centrifuged and stored frozen at -20°C.

Cell line and cell culture

HCT116 colon carcinoma cells (male) and A549 lung adenocarcinoma cells (male) were obtained from ATCC and supplied mycoplasma free. HCT116 cells were maintained in McCoy's 5A (1x) + Glutamax-I growth medium (Gibco, 36600-021) supplemented with fetal bovine serum (FBS, Gibco Life Technologies, 10270-106) at a final concentration of 10%. A549 cells were cultured in Dulbecco Modified Eagle medium (DMEM) (1x) +Glutamax-I (Gibco Life Technologies, 31966-021) with 10% FBS. HeLa Kyoto cells (female, from the laboratory of Jonathan Pines, Institute of Cancer Research, London, U.K.), EUFA423 cells (male, from the European Fanconi Anemia Registry, VU University Medical Center, Amsterdam) and EUFA423+BRCA2 cells (Hattori et al., 2011) were cultured in DMEM with 10% FBS. All cells were grown at 37°C/5% CO_2 in a humidified environment and all the assays were performed using these culturing conditions.

METHOD DETAILS

Chemical synthesis

See Methods S1 for synthetic methods for all compounds.

Protein production

All recombinant proteins used in this study (various HumRadA mutants and ChimRAD51) as well Alexa Fluor 488-labelled BRC repeat were prepared as described in detail in (Moschetti et al., 2016). The humanised RadA mutants, corresponding to residues 108-350 of *Pyrococcus furiosus* RadA, were expressed in BL21(DE3) strain of *E. coli* from T7-promoter driven plasmid pBAT4. Cells were grown in 2YT medium at 37°C until OD_{600} of 0.8-1.0 after which the expression was induced with 400 uM IPTG. Cells were grown for further 3 hours, centrifuged and stored frozen at -20°C. Thawed cells were lysed in 20 mM MES, 0.5 mM EDTA pH 6.0 buffer using Emulsiflex C5 homogeniser. The lysate was heated to 65°C for 10 minutes to denature most of the *E. coli* protein. The sample was centrifuged for 20-30 min at 15,000 xg and the soluble fraction from 1 liter of expression was loaded into a 5 ml HiTrap SP HP column (Cytiva) equilibrated with the lysis buffer. The bound proteins were eluted with a linear gradient to 0.5 M NaCl in 50 mM MES pH 6.0, with HumRadA variants eluting at around 0.3 M salt. The peak fractions were pooled and concentrated using centrifugal concentrators with MWCO of 10 kDa to 2 ml. The sample was loaded into a Superdex 75 HiLoad 16/600 pg size exclusion column (Cytiva) which was equilibrated with 20 mM MES, 100 mM NaCl, 0.5 mM EDTA, pH 6.0. The samples eluted with an isocratic eluent at the expected position for their monomeric molecular weights as a single peak. The peak fractions were pooled, concentrated as before to typically 0.5 mM concentration (as determined by UV absorbance at 280 nm using calculated molar absorption coefficient for each protein), flash frozen in liquid N_2 in 25 ul aliquots and stored at -80°C.

The chimeric RAD51/RadA protein, ChimRAD51, used in FP assays was expressed as fusion construct containing a His-tag, GST, the BRC4 repeat in which the phenylalanine in the FxxA motif was mutated to alanine, a TEV cleavage site and ChimRAD51. The expression of this protein was carried out as describe above. After lysis of the cells and clarification of the lysate by centrifugation the soluble fraction was loaded into NiSepharose colun (Cytiva) under gravity and eluted after washing with 1000 mM imidazole. The fusion protein was concentrated and purified further using a Superdex 75 HiLoad 16/600 pg size exclusion column (Cytiva) in 50 mM Hepes/Na (pH 8.0), 200 mM KCl, 100 mM arginine, 100 mM glutamate, and 100 mM phenylalanine buffer. Peak fractions were flash frozen in liquid N_2 and stored at -80°C before TEV cleavage. After digestion with TEV the sample was re-purified by size exclusion chromatography using the same conditions, separating the monomeric ChimRAD51 from dimeric GST fusion part. This protein could be stored in the fridge for up to a week for FP assays. For ITC experiments the sample was passed through a Ni-Sepharose column to capture any remaining His-GST fusion partner prior to use.

The labelled BRC4 repeat was synthesized using standard chemistry using Fmoc protection with an additional *N*-terminal cysteine which was used for labelling the peptide using maleimide Alexa Fluor 488 dye (ThermoFisher). The labelled protein was purified by reversed phase chromatography using a 4.6 × 250 mm Ace C18 300 Å column. The peptide was confirmed to be fully-labelled by mass spectrometry and quantified using the molar absorption coefficient for the fluorophore (73,000 mol^{-1} cm^{+1} at 495 nm).

ITC

ITC was performed using a Microcal iTC200 instrument at 25°C. Experiments typically involved titrating a 10-fold excess of ligand in the injection syringe against the protein ([HumRadA2] = 60 μM or [ChimRAD51] = 20 μM) in either 200 mM Tris buffer at pH 7.5 and 100 mM NaCl (HumRadA2) or 20 mM potassium phosphate at pH 8.0 and 192 mM KCl (ChimRAD51). Titrations were typically performed with 5-10% DMSO and care was taken to ensure that the DMSO concentrations in the protein and ligand solutions were well matched. The raw ITC data were fitted using a single-site binding model in Microcal ITC LLC data analysis program in the Origin 7.0 package to derive the dissociation constant, stoichiometry and ΔH of binding. For low affinity fragments stoichiometry was fixed to 1:1.

FP assay

Fluorescence Polarisation (FP) competition experiments were performed as described in (Moschetti et al., 2016). In brief, binding of 10 nM Alexa Fluor 488-labelled BRC4 peptide to 50 nM ChimRAD51 protein (giving approximately 80-90% saturation of binding) was competed with increasing concentration of inhibitor and the resulting competitive binding isotherms were measured and fitted using the expression described by (Wang, 1995) using Pro Fit software package (Quan Soft).

X-ray crystallography

Crystallisation and structure determination was done as described in detail previously (Moschetti et al., 2016) using proteins produced in *E. coli,* as described above. Ligands were soaked into HumRadA1 or HumRadA22F crystals in the presence of cryo-protectant typically overnight and crystals cryo-cooled in liquid N_2. Diffraction data was collected at Diamond and ESRF synchrotrons and processed with XDS or autoproc (Kabsch, 2010; Vonrhein et al., 2011). Structures were solved by molecular replacement using corresponding apo structures and ligands fitted into the emerging density after brief refinement and complex structures refined to completion using phenix.refine or autoBuster (Adams et al., 2010).

Immunofluorescent visualisation of RAD51 foci/γH2AX foci in A549 cells using the Cellomics Arrayscan V^{ti} high content microscopy

A549 cells were seeded at 15000 cells/well in 100 μl (1.5x10^5 cells/ml) in Nunc 96-well plates (cat# 167008) and grown overnight prior to the drug treatment. Compounds were added to cells such that the final DMSO concentration did not exceed 1% v/v. Following compound addition, cells were exposed with specified levels of ionising radiation using the Xstrahl RS225 X-ray generator. After incubation with the compound for 6 hours, the medium was removed by aspiration and the cells washed twice in 1xPBS. Cells were fixed using fixative solution (4% formaldehyde diluted in PBS) pre-warmed to 37°C for 10 min at room temperature. Cells were then washed twice in 100 μl 1x PBS at room temperature. Cells were then incubated in 100μl permeabilisation buffer for 5 minutes at room temperature after which they were incubated with 100μl of blocking buffer (2% BSA (w/v), 0.2% Tween 20 (v/v), 0.1% Triton X-100 (v/v) in PBS) for 90 minutes at room temperature. Cells were subsequently incubated with 50 μl of mouse polyclonal anti-RAD51 Antibody (Abnova, cat # H00005888-B01P) diluted 1:200 in blocking solution for 2 h at room temperature. Cells were washed in 100 μl wash buffer at room temperature (0.2% Tween 20 (v/v), 0.1% Triton X-100 (v/v) in 1x PBS) then incubated in 50 μl Alexa Fluor 488 labelled anti-mouse secondary antibody (1:500) and Hoechst 33342 (10 mg/ml stock) counterstain at 1:1000 in blocking solution for 60 minutes at room temperature. Finally, cells were washed twice in wash buffer and then twice in PBS and then stored in 100 μl in PBS with a light protective seal at 4°C until read on the Cellomics Arrayscan V^{ti} using a spot detector protocol. The number of cells analysed was 800 and the parameter used for analysis was Total Spot Area.

For detection of γH2AX foci in A549 cells, 10,000 cells/well (1x10^5 cells/ml) were seeded in 100 μl and left to grow overnight before treatment with compound. Cells were subsequently exposed to compounds and either 3 Gy ionising radiation or mock treated (left on the bench at room temperature). Staining protocol was identical as for RAD51 foci but anti-phospho γH2AX primary mouse monoclonal antibody was used (Millipore, cat#05-636) at 1:2000 dilution. Image analysis was done using Cellomics software.

SRB growth inhibition assay

Adherent cell lines (HCT116 and A549 cells) were seeded into flat-bottomed tissue culture 96-well plates in a volume of 150 μL of growth medium. HCT116 cells were seeded at 750 cells per well and A549 cells were seeded at 1000 cells per well. After 24 hours, compounds dissolved in DMSO were diluted in growth medium and were added to cells such that the final DMSO concentration was 1% (v/v) and the final volume in the well was 200 μL. Cells were then incubated in the presence of compound for 96 hours before fixation.

Medium was removed from cells and 100 μL of cold 1% (v/v) trichloroacetic acid was added for 30 minutes at 4 degrees. The plates were washed three times in tap water and left to dry at room temperature. The fixed cells were stained in a 0.057% sulphorodamine B/ 1% acetic acid solution (w/v) and incubated at room temperature with agitation for 30 minutes after which the dye was removed and the plates washed in 1% (v/v) acetic acid and left to dry. The dye was then solubilised in 10 mM Tris solution (pH8) and incubated for 30 minutes under agitation. The plates were then read on a PHERAstar plus plate reader (BMG Labtech) using the fluorescence intensity module (540-590 nm). Growth inhibition was calculated relative to DMSO controls and GI_{50} values were calculated using Graphpad Prism.

For the PARP inhibitor experiments, the SRB method was used as described above to measure growth inhibition with the exception that cells were seeded into 150 μl medium and then a combination of either 25 μl of CAM833, AZD2461 (SelleckChem; #S7029) or DMSO was added to give a total volume of 200 ul in the well.

Flow cytometry

Propidium iodide staining solution (PI solution) was used at the following final concentrations: 200 ug/ml RNAase A (Sigma Aldrich, cat# 10109169001), 0.1% Triton-X 100 (v/v) and 20 ug/ml of propidium iodide solution diluted in 1x PBS. HCT116 cells were grown in 6-well plates in a total volume of 2 ml and treated with either test compound or DMSO control for the designated time. After treatment, medium was collected from the cells which were then washed in 1x PBS then removed from the plastic by the addition of in 500 μl Trypsin/EDTA until cells were monodispersed. The trypsin was neutralised by the removed media and the cell suspension was spun at 1000 rpm for 5 minutes. Cells were then washed a further time in ice cold 1xPBS and spun at 1000 rpm for 5 minutes. Cells were then fixed in 4.5ml 70% ice cold ethanol and 0.5 ml ice cold 1xPBS. Cells were left in fixing solution overnight at 4°C until processing. Cells were spun at 1000 rpm for 5 mins and then washed in 1xPBS, re-suspended in 0.5-1 ml of the PI solution at incubated in the dark for 2 hours at room temperature. Cells were then counted and analysed using a Becton Dickinson LSR II cytometer and FCS Express software.

Super-resolution microscopy

Single Molecule Localization Microscopy (SMLM) was achieved by direct Stochastic Optical Reconstruction Microscopy (d-STORM) as described (Haas et al., 2018). Briefly, samples were prepared for one colour 2D d-STORM utilizing a buffer containing 100 mM MEA-HCL (Sigma, M6500), 10% glucose (Sigma), 0.5 mg/ml glucose oxidase (Sigma, G2133) and 40 μg/ml catalase (Sigma, C100) in water at pH 7.5. Samples were imaged by direct STORM at room temperature in sealed 8-well ibidi μ-slides utilizing an inverted N-STROM microscope (Nikon Ti, Japan) equipped with an Apochromat 100x/1.49 NA oil immersion objective. Samples were let to equilibrate for at least 30 minutes before imaging to minimize thermal drift. Images were then acquired with highly inclined illumination and focus was maintained by hardware autofocusing (Nikon Perfect Focus System). AlexaFluor647 was first pumped in its dark state using the 640 nm laser line at maximum power (~150 mW) and then imaged continuously with a power density of ~3 kW/cm^2. Data were acquired in 'streaming mode' with a field-of-view (FOV) of 256x256 pixels (160 nm pixel size), at 65 frames per second for 25,000 frames with an EMCCD camera (iXon Ultra DU897, Andor). The sparsity of single molecules per frame was controlled using ~0.6 mW of the 405 nm laser. Images of AlexaFluor647 were acquired with a Quad Band Set for TIRF applications (Chroma, TRF89901, ET – 405/488/561/640 nm) and the ET645/75 m emission filter (Chroma).

Cluster data analysis

Single molecule data was analysed using the Grafeo program available at https://github.com/inatamara/Grafeo-dSTORM-analysis-, as described in (Haas et al., 2018). Briefly, all localizations with fewer than 1,000 detected photons or localization precision lower than 20 nm were discarded. Next, the data was filtered using 2D Voronoi diagrams, setting the minimum density (an inverse of Voronoi polygon VP size) to 5*10^{-5} nm^{-2}. Finally, small isolated detections were supressed by thresholding univariate distance distribution function – a detection was rejected if it had less than 20 neighbours at the distance ≤100 nm. Next, two-dimensional Delaunay triangulation (DT) was computed. Localizations were assigned to discreet clusters, connected components, by removing all DT edges larger than 20 nm. All segmented connected components having less than 3 localizations were discarded. The number of RAD51 molecules inside a cluster was estimated by dividing the number of localization within a cluster by the expected number of localization obtained from isolated secondary antibodies used to label RAD51.

mClover Lamin A HDR assay

CAM833 was added to HeLa Kyoto cells grown on coverslips in 6 well plates 1 hour before transfection. Cells were transfected with sgRNA plasmid targeting Lamin A (pUC CBA-SpCas9.EF1a-BFP.sgLMNA, Addgene Plasmid #98971) and donor plasmid (pCAGGS Donor mClover-LMNA, Addgene Plasmid #98970) using JetPRIME transfection reagent (Polyplus Transfection). The next day, cell culture media were replaced with fresh media containing CAM833. Three days after transfection, cells were fixed with 4% PFA for 10 minutes and permeabilized with TBS-Triton for 5 minutes before mounting. Images acquired with LSM 880 microscope were analyzed with Image J software (Schneider et al., 2012). HDR positive cells were defined as cells with mean mClover nuclear intensity over a threshold set for each experiment (Arnoult et al., 2017; Buisson et al., 2017).

QUANTIFICATION AND STATISTICAL ANALYSES

In the FP competition assay, each competitive binding isotherm resulted from averaging two replicate measurements from the same assay plate and this constituted a single independent measure of the binding isotherm. Per each candidate molecule we collected a minimum of three independently measured isotherms. These isotherms were averaged and then analysed by means of non-linear regression using the expression described by Wang (1995), implemented in Pro Fit software package (Quan Soft), that enabled us to estimate the pKd (the negative of the base-10 logarithm of the equilibrium dissociation constant) and the pKd's standard deviation. These values were used to initially rank compounds. Finally, each pK_d was converted into the corresponding equilibrium dissociation constant by exponentiation. For those candidate molecules that were measured repeatedly and were thus associated to a n>3, before exponentiation we used the pK_d estimates to calculate geometric average and Standard Deviations from the mean.

For microscopy experiments evaluating RAD51 and γ-H2AX foci, analyses were with the Cellomics Arrayscan V^{ti} instrument software, and for mClover Lamin A detection, with ImageJ software (Schneider et al., 2012). Figure 3 shows mean values ±SEM of foci counts. Figure 5 shows the means, errors and tests of significance for mClover Lamin A detection. Details are provided in the Figure legend. For super-resolution microscopy cluster analysis in Figure 4B, simultaneous comparisons of the median values of multiple groups were performed using the Kruskal-Wallis test at the significance level alpha of 0.05 and familywise error rate was corrected by adjusting p-values using the Tukey-Kramer method. Super-resolution image analysis was using the Grafeo program available at https://github.com/inatamara/Grafeo-dSTORM-analysis, as described in (Haas et al., 2018). Details are described in the Figure legend. For the SRB growth inhibition assay in Figures 5E, 6, and 7, GI_{50} values were calculated using Graphpad Prism and are shown as data points and bars representing means and SEMs. Details are described in the Figure legends.

Cell Chemical Biology, Volume *28*

Supplemental information

A small-molecule inhibitor of the BRCA2-RAD51 interaction modulates RAD51 assembly and potentiates DNA damage-induced cell death

Duncan E. Scott, Nicola J. Francis-Newton, May E. Marsh, Anthony G. Coyne, Gerhard Fischer, Tommaso Moschetti, Andrew R. Bayly, Timothy D. Sharpe, Kalina T. Haas, Lorraine Barber, Chiara R. Valenzano, Rajavel Srinivasan, David J. Huggins, Miyoung Lee, Amy Emery, Bryn Hardwick, Matthias Ehebauer, Claudio Dagostin, Alessandro Esposito, Luca Pellegrini, Trevor Perrior, Grahame McKenzie, Tom L. Blundell, Marko Hyvönen, John Skidmore, Ashok R. Venkitaraman, and Chris Abell

Supplementary material

A small-molecule inhibitor of the BRCA2-RAD51 interaction modulates RAD51 assembly and potentiates DNA damage-induced cell death.

Duncan E. Scott, Nicola J. Francis-Newton, May E. Marsh, Anthony G. Coyne, Gerhard Fischer, Tommaso Moschetti, Andrew R. Bayly, Timothy D. Sharpe, Kalina T. Haas, Lorraine Barber, Chiara R. Valenzano, Rajavel Srinivasan, David J. Huggins, Miyoung Lee, Amy Emery, Bryn Hardwick, Matthias Ehebauer, Claudio Dagostin, Alessandro Esposito, Luca Pellegrini, Trevor Perrior, Grahame McKenzie, Tom L. Blundell, Marko Hyvönen, John Skidmore, Ashok R. Venkitaraman, Chris Abell.

Table of Contents

Supplementary material 1

Figure S1. ITC data for the binding of **3** (left, K_d 1.3 mM) and **4** (right, K_d 3 µM) to HumRadA2 (related to figure 2). 2

Figure S2. ITC data for the binding of **5** to HumRadA2 (K_d 220 nM) (related to figure 2). 2

Figure S3. Structure of **6** bound to two forms of humanised RadA (related to figure 2). 3

Figure S4. Inhibition of RAD51 oligomerisation by CAM833 (related to figure 2). 3

Figure S5. Structure of **CAM833** bound to HumRadA22F (related to figure 2). 4

Table S1. Calculated and measured ADMET and developability properties for **CAM833** (related to figure 2 and STAR methods). 5

Table S2. CAM833 growth inhibition data for a range of cancer-derived human cell lines (related to figure 5). 6

Table S3 Crystallographic data collection and refinement statistics (related to figures 2, S3 and S5). 7

Methods S1 – chemical synthesis (related to figure 2). 8

Solvents and Reagents 8

Chromatography 8

Nuclear Magnetic Resonance Spectroscopy 8

Abbreviations 8

LCMS 8

Synthetic methods 8

General procedure A 8

General procedure B 9

(*S*)-1-((2-Naphthoyl)-L-alanyl)-*N*-((*S*)-1-phenylethyl)pyrrolidine-2-carboxamide (5) 9

N-(2-((*S*)-2-(((*S*)-1-(4-methoxyphenyl)ethyl)carbamoyl)pyrrolidin-1-yl)-2-oxoethyl)quinoline-2-carboxamide (6) 10

N-(2-((2*S*,4*R*)-2-(((*S*)-1-(2-chloro-4-methoxyphenyl)ethyl)carbamoyl)-4-hydroxypyrrolidin-1-yl)-2-oxoethyl)-6-fluoroquinoline-2-carboxamide CAM833A 11

Figure S1. ITC data for the binding of **3** (left, K_d 1.3 mM) and **4** (right, K_d 3 µM) to HumRadA2 (related to figure 2)

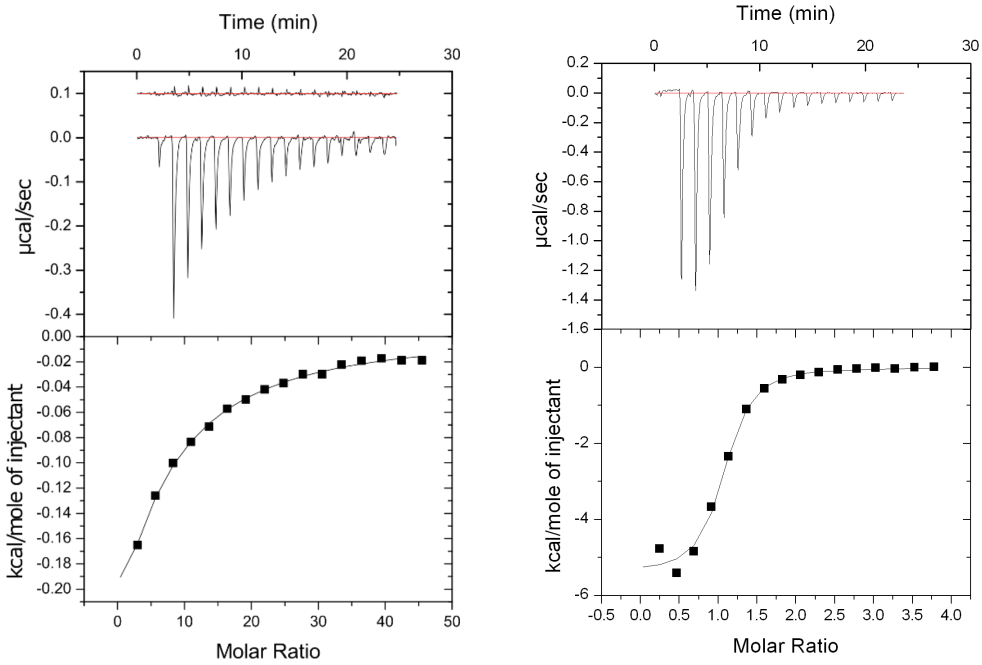

Figure S2. ITC data for the binding of **5** to HumRadA2 (K_d 220 nM) (related to figure 2).

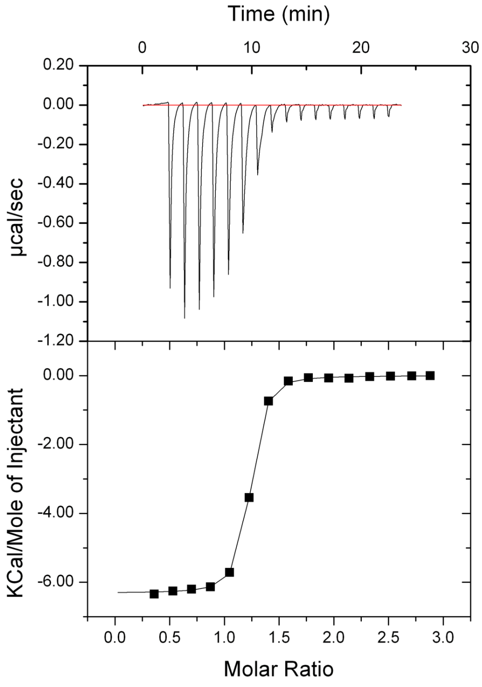

Figure S3. Structure of **6** bound to two forms of humanised RadA (related to figure 2).

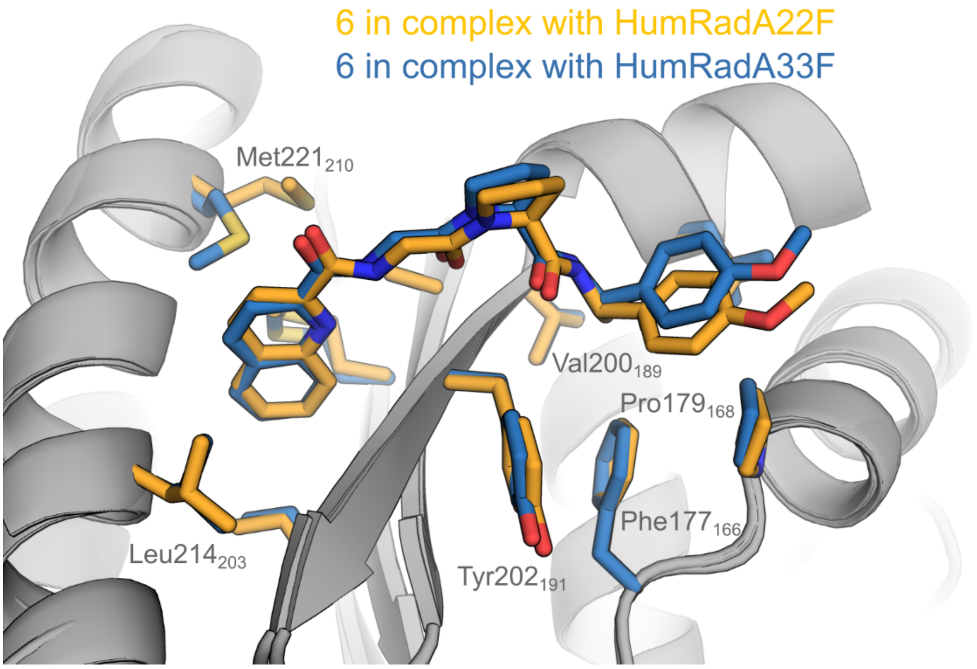

HumRadA22F (orange carbons, PDB:6TW4) and HumRadA33F (blue carbons, PDB: 6XTW) in complex with **6** with the side chains of residues around the Phe and Ala pockets shown as sticks and in the same colour as the ligand bound to that protein.

Figure S4. Inhibition of RAD51 oligomerisation by CAM833 (related to figure 2).

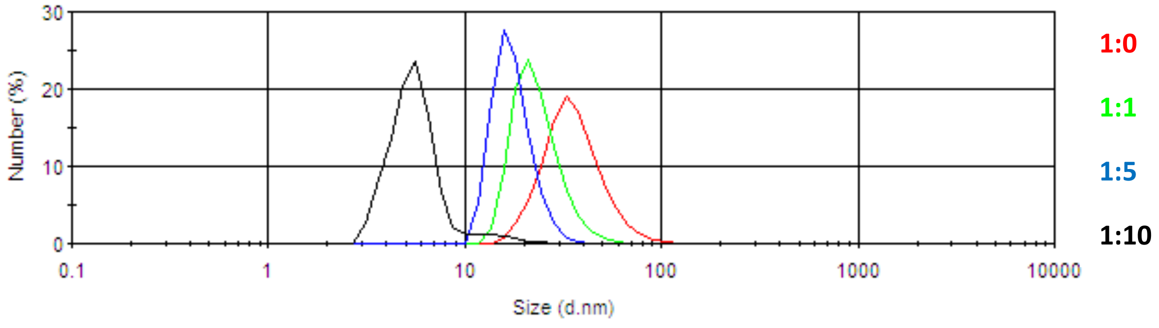

Particle size distribution (in nm) of full-length RAD51 protein in the absence (red line) and presence of increasing concentrations of CAM833. Green line indicates 1:1 stoichiometry of RAD51:CAM833, blue line is for sample with 1:5 stoichiometry and the black line for 1:10 stoichiometry of the components.

Figure S5. Structure of **CAM833** bound to HumRadA22F (related to figure 2).

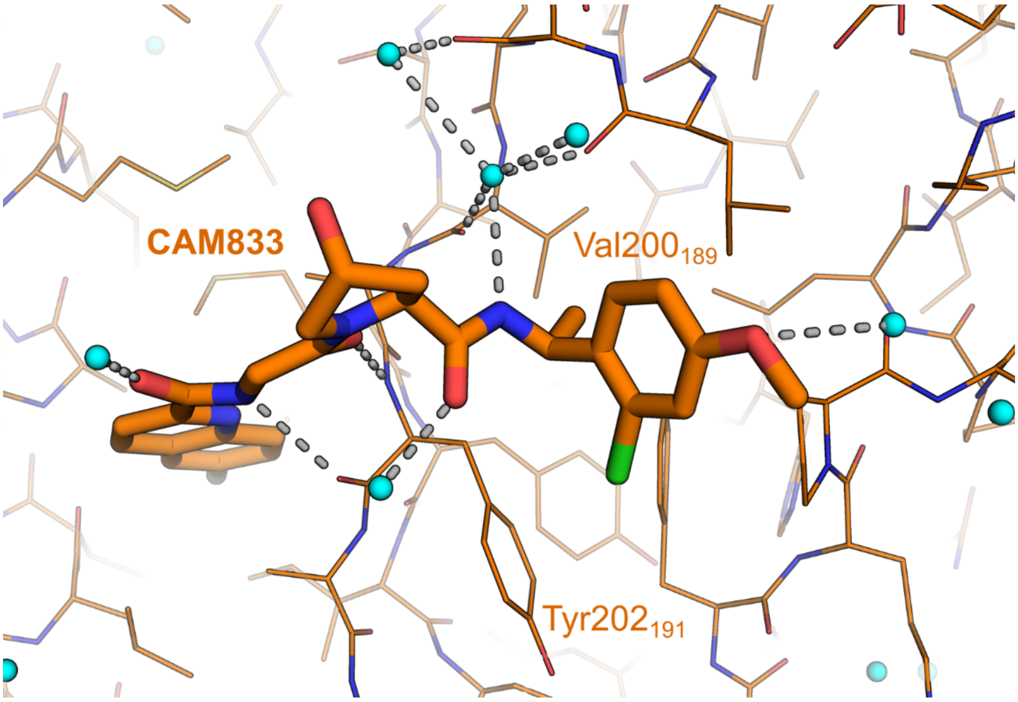

CAM833, in thicker sticks, is bound to HumRadA22F with hydrogen bonds between the inhibitor and the protein (thinner lines) or waters (small cyan spheres) shown as grey dotted lines.

Table S1. Calculated and measured ADMET and developability properties for **CAM833** (related to figure 2 and STAR methods).

MW	529
PSA	120
clogP[a]	2.73
K_D FP (ChimRAD51)	355 nM,[b] (n=8)
Aqueous solubility [c]	7.75 µg/ml (n=2, s.d. 0.549)
Microsomal CL_{int} [d]	Mouse $t_{½}$ 43.1 min<br>32.2 ± 2.99 µL/min/mg protein<br>Rat $t_{½}$ 47.9 min<br>28.9 ± 4.16 µL/min/mg protein<br>Human $t_{½}$ 76.8 min<br>18 ± 4.52 µL/min/mg protein
CYP450 IC_{50} (1A2, 2C9, 2C19, 2D6, 3A4 midazolam and testosterone sites)	all > 25 µM
hERG electrophysiology @ 10 uM[e]	12%
Human plasma protein binding [f]	99.2% bound
Caco-2 permeability<br>AtoB P_{app} (10^{-6} cm s^{-1}) / Efflux Ratio	0.23 / 195
Selectivity Cerep ExpresSPanel	no inhibition of binding >50% at 10 µM
Mouse iv pharmacokinetics at 1 mg/kg[g]	t½ = 1.44 h (n=2)<br>CL 44.6 ml/min/kg (n=3)<br>Vd_{ss} 2.51 L/kg (n=2)
Mouse oral screen at 50 mg/kg [h]	Cmax 8323 ng/ml (n=3)<br>F = 44%

a. Calculated using Chemdraw 16

b. pK_d = 6.45 ± 0.16, n=8 determined by FP

c. Thermodynamic solubility from solid, determined overnight in pH 7.4 buffer.

d. Intrinsic clearance calculated from 5 timepoints over a 45 minute experiment reported as half-life and intrinsic clearance ± standard error

e. Inhibition of hERG tail-currents measured by whole-cell voltage-clamping in mammalian cells.

f. Determined by equilibrium dialysis

g. Fasted male CD-1 mice. 0.5 mg/mL in 20%HP-β-CD in water, clear solution. n=3 or 2

h. Fasted male CD-1 mice, fasted. 5 mg/mL in 70% PEG400 / 30% water, clear solution. n=3 or 2.

Table S2. CAM833 growth inhibition data for a range of cancer-derived human cell lines (related to figure 5).

Cell Line	Absolute EC_{50} (µM)
HUVEC	133.0
Mia-pa-ca2	68.6
NCI-H209	69.3
OVCAR3	53.6
PC3	91.2
SK-MEL-24	143.5
U20S	40.5
BT549	73.8
HCT116	63.5
A375	72.0
BT20	61.9
HT1367	104.0
MDA-MB-453	48.0
RT112/84	70.6
T.Tn	55.8
U118MG	90.3
SCaBER	47.0
SK-OV-3	104.8
T24/83	96.7
U87	108.8
A549	39.0
Calu3	83.3
HepG2	68.1
HFL-1	98.8
HL-60	87.7
K562	137.1
LN229	117.3
Raji	95.6
RKO	63.5
5637.00	84.5
A2780	46.5
Caki1	73.0
PANC1	96.0
SW756	124.3

Table S3 Crystallographic data collection and refinement statistics (related to figures 2, S3 and S5).

Ligand	**3**	**4**	**6**	**6**	**CAM833**
Protein form	HumRadA1	HumRadA1	HumRadA22F	HumRadA33F	HumRadA22F
PDB code:	6TV3	6TWR	6TW4	6XTW	6TW9
**Data Collection and Processing:**					
Synchrotron beamline	ESRF ID14-4	DLS I04	DLS I24	DLS I03	DLS I02
Wavelength (Å)<br>Resolution range (Å)<br>(High resolution bin)	0.9795<br>21.03 - 1.50<br>(1.59 - 1.50)	0.9702<br>31.63 - 1.35<br>(1.43 - 1.35)	0.9686<br>40.39 - 1.73<br>(1.77 - 1.73)	0.9300<br>100.82-2.31<br>(2.43-2.31)	0.9795<br>61.21 - 1.52<br>(1.522 - 1.517)
Space group	P 1 21 1	P 21 21 21	P 21 21 21	P31 2 1	P 21 21 21
Unit cell (a b c) (Å)	37.67 79.24 39.43	40.53 61.95 87.72	40.39 60.15 88.20	89.83 89.83 100.82	40.38 61.21 87.68
Unit cell (α β γ) (°)	90.00 118.18 90.00	90.00 90.00 90.00	90.00 90.00 90.00	90.00 90.00 120.00	90.00 90.00 90.00
Total number of reflections	104241 (31919)	325400 (44541)	182953 (12814)	107445 (16048)	149322 (1612)
Number of unique reflections	31919 (5063)	48841 (7575)	23054 (1639)	21180 (3039)	32571 (351)
Multiplicity	3.7 (3.6)	6.7 (5.9)	7.9 (7.8)	5.1 (5.3)	4.6 (4.6)
Completeness (%)	97.4 (96.4)	99.5 (96.9)	99.6 (98.6)	99.9 (99.9)	94.6 (97.5)
Mean I/sigma(I)	15.9 (2.9)	13.8 (2.2)	15.2 (0.6)	14.4 (2.8)	12.5 (2.2)
R_{merge}	0.049 (0.45)	0.080 (0.64)	0.087 (1.25)	0.067 (0.621)	0.087 (0.67)
R_{pim}	- (-)	- (-)	0.035 (0.50)	0.036 (0.330)	0.043 (0.34)
CC-half	0.999 (0.87)	0.998 (0.77)	- (-)	- (-)	- (-)
**Refinement:**					
R / R_{free}	0.183 / 0.217	0.187 / 0.210	0.178 / 0.210	0.184 (0.229)	0.156 / 0.172
Number of atoms	1985	2334	1955	3664	3853
No. of ligand atoms	40	75	35	35	44
No. of waters	221	358	143	118	258
No. of protein residues	219	227	226	227	224
Average/Wilson<br>B-factor (Å^2)	24.5 / 18.5	18.2 / 19.3	27.8 / 23.2	48.0	17.0 / 15.2
B-factor for ligands (Å^2)	34.8	26.4	28.6	52.0	19.1
B-factor for solvent (Å^2)	39.3	29	39	46.9	30.6
RMS (bonds) (Å)	0.01	0.005	0.01	0.007	0.011
RMS (bond angles)	1.12	0.81	1.09	0.862	1.16

Methods S1 – chemical synthesis (related to figure 2).

Solvents and Reagents

Unless otherwise stated starting materials and reagents were purchased from regular suppliers. Dry solvents were purchased and used as provided.

Chromatography

Thin layer chromatography (TLC) was performed on glass plates coated with Merck 60 F254 silica and visualization was achieved by UV light or by staining potassium permanganate. Flash column chromatography was using a Biotage Isolera One and Biotage Isolera Four systems with UV detection at 254 nm and 280 nm and commercially available cartridges.

Nuclear Magnetic Resonance Spectroscopy

^{1}H NMR spectra were recorded on a Bruker Avance 400 (400 MHz), or Bruker Avance Cryo 500 (500 MHz). Chemical shifts are quoted in ppm and are referenced to the residual non-deuterated solvent peak, and are reported (based on appearance rather than interpretation) as follows: chemical shift δ /ppm (multiplicity, coupling constant J/Hz, number of protons) [br, broad; s, singlet; d, doublet; t, triplet; q, quartet; qui, quintet; sept, septet; m, multiplet]. All J values are given in Hz. Fractional integrations are reported where conformational restriction of peptidic compounds results in separate signals on the nmr timescale.

Abbreviations

DCM - dichloromethane
DMAP – 4-dimethylaminopyridine
EDAC - *N*-(3-dimethylaminopropyl)-*N′*-ethylcarbodiimide hydrochloride
HBTU - *N,N,N′,N′*-tetramethyl-*O*-(1*H*-benzotriazol-1-yl)uronium hexafluorophosphate
PyBOP - benzotriazole-1-yl-oxy-tris-pyrrolidino-phosphonium hexafluorophosphate
THF – tetrahydrofuran

LCMS

High-resolution mass measurements were performed on a Waters LCT Premier mass spectrometer or a Kratos Concept mass spectrometer. Low-resolution measurements were recorded on a Waters / ZQ LCMS and on a Waters Acquity UPLC HClass LCMS. All final compounds used for screening and cell experiments were at least 95% pure as determined by LCMS unless otherwise stated.

Synthetic methods

Tetrapeptide (**1**) was prepared as described previously (Scott et al., 2016). Fragments 2-naphthol (**2**) and 3-amino-2-naphthoic acid (**3**) are commercially available and used as supplied. Tetrapeptide **(4)** was prepared using standard solid phase chemistry with Fmoc protection by the Protein and Nucleic Acid Service at the Department of Biochemistry (University of Cambridge).

General procedure A

EDAC (1.5 equiv.) was added to a stirred solution of acid (1 equiv.), amine (2 equiv.), *N*-methylmorpholine (3 equiv.) and DMAP (1 equiv.) in DCM (0.05 M) and stirred until the reaction was judged complete. The solvent was removed *in vacuo* and DCM and H_2O were added to the residue, the organic layer was separated, washed with H_2O, brine, dried with $MgSO_4$, filtered and the solvent removed *in vacuo*. The residue was purified by flash chromatography (FC) (2-20% MeOH: DCM) to give the product.

General procedure B

To a solution of acid (1 equiv.), amine (1-1.1 equiv.) and DIPEA (2-5 equiv.) in solvent was added PyBOP (1.1-1.4 equiv.) and the reaction was stirred until complete and then concentrated *in vacuo*. Unless stated otherwise, the crude was diluted with ethyl acetate and the organics were washed three times with water, and then brine and dried before purifying typically by FC as described to give the product.

(*S*)-1-((2-Naphthoyl)-L-alanyl)-*N*-((*S*)-1-phenylethyl)pyrrolidine-2-carboxamide (5)

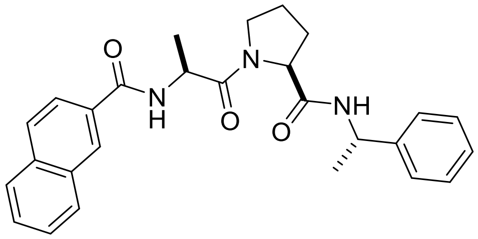

Methyl (2-naphthoyl)-L-alanyl-L-prolinate
A mixture of methyl L-alanyl-L-prolinate hydrochloride (200 mg, 0.84 mmol), 2-naphthoic acid (218 mg, 1.27 mmol), EDAC (243 mg, 1.27 mmol), *N*-methylmorpholine (204 µL, 1.86 mmol) and DMAP (103 mg, 0.84 mmol) in DCM (10 mL) was stirred until judged complete and purified by FC (1-10% MeOH: DCM) to give the product as a white foam (240 mg, 81%).
^{1}H NMR (500 MHz, Methanol-d_4) δ 8.43 (d, *J* = 1.6 Hz, 1H), 7.98 (dd, *J* = 7.8, 1.6 Hz, 1H), 7.96 – 7.88 (m, 3H), 7.62 – 7.54 (m, 2H), 4.52 (dd, *J* = 8.6, 4.7 Hz, 1H), 3.94 (dt, *J* = 9.9, 7.1 Hz, 1H), 3.75 (dt, *J* = 10.1, 6.6 Hz, 1H), 3.72 (s, 3H), 2.36 – 2.24 (m, 1H), 2.09 (p, *J* = 6.7 Hz, 2H), 1.99 (dtd, *J* = 12.5, 6.7, 4.8 Hz, 1H), 1.51 (d, *J* = 7.1 Hz, 3H). Proline α-CH is under the solvent peak (4.83-4.88 ppm)
LCMS m/z 355.3 $(M+H)^+$

(2-Naphthoyl)-L-alanyl-L-proline
To a solution of methyl (2-naphthoyl)-L-alanyl-L-prolinate (230 mg, 0.65 mmol) in THF:H_2O (1:1, 20 mL) was added dropwise at 0 °C a solution of NaOH (104 mg, 2.6 mmol) in H_2O (1 mL). The reaction mixture was stirred for 10 mins at 0 °C, allowed to warm to rt and stirred for 2 hours. The mixture was concentrated *in vacuo*, acidified on ice to pH 2 and the resulting white suspension was extracted with DCM (4 x 100 mL). The organic extracts were combined, dried with $MgSO_4$, filtered and the solvent removed *in vacuo* to give the acid as a white foam (212 mg, 96%) which was used without further purification.
^{1}H NMR (400 MHz, Chloroform-*d*) δ 8.30 – 8.24 (m, 0.85H), 8.23 (d, *J* = 1.3 Hz, 0.15H), 7.88 – 7.72 (m, 4H), 7.61 (d, *J* = 7.6 Hz, 0.15H), 7.53 – 7.43 (m, 2H), 7.38 (d, *J* = 7.6 Hz, 0.85H), 4.98 (p, *J* = 7.0 Hz, 0.85H), 4.85 (p, *J* = 6.8 Hz, 0.15H), 4.56 (dd, *J* = 7.0, 5.7 Hz, 0.85H), 4.49 (dd, *J* = 7.5, 3.2 Hz, 0.15H), 3.84 – 3.46 (m, 3H), 2.24 (td, *J* = 7.1, 3.1 Hz, 0.15H), 2.17 – 2.11 (m, 1H), 2.07 – 1.93 (m, 1.7H), 1.90 – 1.81 (m, 0.15H), 1.81 – 1.75 (m, 1H), 1.46 (d, *J* = 6.9 Hz, 2.55H), 1.42 (d, *J* = 6.7 Hz, 0.45H).
LCMS m/z 341.2 $(M+H)^+$

(*S*)-1-((2-Naphthoyl)-L-alanyl)-*N*-((*S*)-1-phenylethyl)pyrrolidine-2-carboxamide (5)
(2-Naphthoyl)-L-alanyl-L-proline (25 mg, 0.07 mmol) was dissolved in DCM (2 mL) and cooled to 4 ˚C under N_2 gas. To the stirred solution was added EDAC (21 mg, 0.11 mmol), (S)-α-methylbenzylamine (19 µL, 0.15 mmol) and DMAP (10 mg, 0.07 mmol). The reaction was stirred for 3 hours, and the solvent was removed *in vacuo.* The residue was dissolved in ethyl acetate (100 ml), washed with water

(2 x 100 ml), washed with brine and then dried ($MgSO_4$) and the solvent was removed *in vacuo*. The crude product was purified by FC (1-10% MeOH: DCM) to give the product as a clear oil (18 mg, 54%).
^{1}H NMR (400 MHz, Chloroform-*d*) δ 8.27 (s, 1H), 7.90 – 7.82 (m, 1H), 7.82 – 7.77 (m, 3H), 7.48 (ddt, *J* = 8.0, 6.9, 5.3 Hz, 2H), 7.33 – 7.13 (m, 6H), 7.04 (d, *J* = 8.1 Hz, 1H), 5.06 – 4.87 (m, 2H), 4.54 (dd, *J* = 8.2, 2.8 Hz, 1H), 3.71 (td, *J* = 9.1, 8.6, 7.2 Hz, 1H), 3.59 (ddd, *J* = 9.8, 8.0, 4.1 Hz, 1H), 2.37 – 2.24 (m, 1H), 2.22 – 2.07 (m, 1H), 2.05 – 1.89 (m, 1H), 1.91 – 1.74 (m, 1H), 1.46 (d, *J* = 6.8 Hz, 3H), 1.39 (d, *J* = 6.9 Hz, 3H).
LCMS m/z 442.1 $(M-H)^-$

N-(2-((*S*)-2-(((*S*)-1-(4-methoxyphenyl)ethyl)carbamoyl)pyrrolidin-1-yl)-2-oxoethyl)quinoline-2-carboxamide (6)

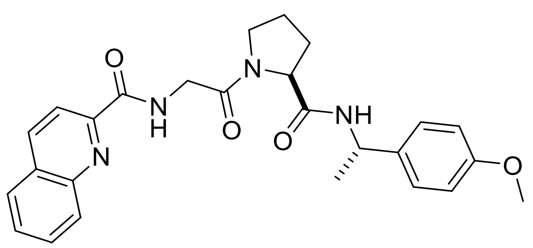

(Quinoline-2-carbonyl)glycyl-L-proline
To a mixture of (quinoline-2-carbonyl)glycine (1.9 g, 8.26 mmol) and L-proline-t-butyl ester (1.71 g, 10.0 mmol) in DCM (15 ml) was added PyBOP (4.5 g, 8.65 mmol) and DIPEA (2.24 ml, 16.5 mmol). The reaction was stirred at rt for 16 hours and then washed with aq. sodium bicarbonate, dried and evaporated. Purification by FC (SiO_2, ethyl acetate/MeOH) afforded the intermediate ester (2.68 g). To a stirred solution of the ester (1.3 g) dissolved in DCM (7 ml) at 0 ˚C was added tri-isopropylsilane (0.1 ml) and then TFA (10 ml). The mixture was stirred for four hours. The solvent was evaporated and the mixture was partitioned between ethyl acetate and 0.8 M aq HCl. The organics were separated, dried and evaporated to generate the product (800 mg, 61% over two steps).
^{1}H NMR (400 MHz, Chloroform-*d*) δ 9.33 (s, 0.2H), 9.05 – 8.96 (m, 0.8H), 8.37 – 8.23 (m, 2H), 8.22 – 8.14 (m, 1H), 7.94 – 7.85 (m, 1H), 7.79 (ddd, *J* = 8.5, 6.9, 1.5 Hz, 1H), 7.69 – 7.60 (m, 1H), 4.76 – 4.67 (m, 0.8H), 4.63 – 4.56 (m, 0.2H), 4.54 – 4.24 (m, 2H), 3.86 – 3.53 (m, 2H), 2.49 – 1.91 (m, 4H).
LCMS m/z 328.1 $(M+H)^+$

***N*-(2-((*S*)-2-(((*S*)-1-(4-methoxyphenyl)ethyl)carbamoyl)pyrrolidin-1-yl)-2-oxoethyl)quinoline-2-carboxamide (6)**

Methoxy-(*S*)-α-methylbenzylamine (45 µL, 0.31 mmol) was coupled to (quinoline-2-carbonyl)glycyl-*L*-proline (50 mg, 0.15 mmol) according to the general procedure A to give the product as a clear oil (39 mg, 55%).
^{1}H NMR (400 MHz, Chloroform-*d*) δ 9.01 – 8.90 (m, 1H), 8.31 (d, *J* = 8.4 Hz, 1H), 8.25 (d, *J* = 8.4 Hz, 1H), 8.19 (dq, *J* = 8.6, 0.9 Hz, 1H), 7.90 (dd, *J* = 8.1, 1.5 Hz, 1H), 7.79 (ddd, *J* = 8.4, 6.9, 1.5 Hz, 1H), 7.65 (ddd, *J* = 8.2, 6.9, 1.2 Hz, 1H), 7.28 – 7.23 (m, 2H), 7.18 (d, *J* = 7.8 Hz, 1H), 6.86 – 6.79 (m, 2H), 5.03 (p, *J* = 7.1 Hz, 1H), 4.62 (dd, *J* = 8.2, 2.2 Hz, 1H), 4.35 (t, *J* = 4.8 Hz, 2H), 3.73 (s, 4H), 3.57 (td, *J* = 9.4, 7.0 Hz, 1H), 2.45 – 2.36 (m, 1H), 2.31 – 2.16 (m, 1H), 2.12 – 2.02 (m, 1H), 1.99 – 1.87 (m, 1H), 1.49 (d, *J* = 7.0 Hz, 3H).
LCMS *m/z* 461.4 $(M+H)^+$

N-(2-((2*S*,4*R*)-2-(((*S*)-1-(2-chloro-4-methoxyphenyl)ethyl)carbamoyl)-4-hydroxypyrrolidin-1-yl)-2-oxoethyl)-6-fluoroquinoline-2-carboxamide CAM833A

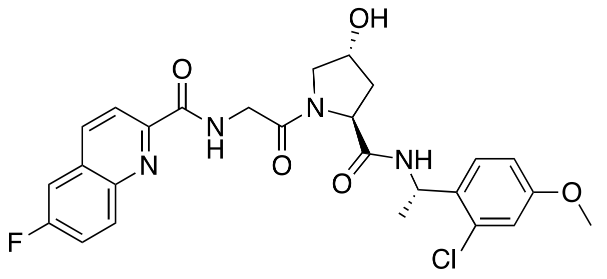

(*S*)-1-(2-Chloro-4-methoxyphenyl)ethan-1-amine

2-Chloro-4-methoxybenzyaldehyde (3.4 g, 19.9 mmol) and (*R*)-(+)-t-butanesulfinamide (2.4 g, 19.9 mmol) were added to THF (100 mL) and $Ti(OEt)_4$ (10.0 g, 43.8 mmol) was added. The reaction was heated at reflux overnight under N_2, cooled to room temperature and brine (100 mL) and ethyl acetate (100 mL) added. The mixture was filtered through celite, washing the celite with ethyl acetate (100 mL). The separated organic layer was dried (Na_2SO_4), filtered, and the solvent removed in vacuo to give the sulfinimide as a white solid (4.9 g, 90%). LCMS *m/z* 274.2 $(M+H)^+$

A portion of sulfinimide (2.5 g, 9.1 mmol) was dissolved in DCM (60 mL) and cooled to -50 ˚C. MeMgBr (6.0 mL, 3.2 M in 2-methyl THF, 19.2 mmol) was added dropwise and the reaction mixture allowed to warm to rt overnight. Saturated aq. NH_4Cl (50 mL) was added, mixture was stirred for 5 mins and extracted with DCM (2 x 50 mL). The combined organic layers were dried through a hydrophobic frit and purified by FC (SiO_2, 12-100% ethyl acetate:pet ether 40-60) to give the sulfinamine as a clear oil (2.5 g, 94%). The diastereomeric ratio following the Grignard addition step was 98:2 as determined by ^1H NMR.

^1H NMR (400 MHz, Chloroform-*d*) δ 7.31 (d, *J* = 8.7 Hz, 1H), 6.89 (d, *J* = 2.6 Hz, 1H), 6.80 (dd, *J* = 8.6, 2.6 Hz, 1H), 4.96 (qd, *J* = 6.7, 4.3 Hz, 1H), 3.78 (s, 3H), 1.52 (d, *J* = 6.7 Hz, 3H), 1.19 (s, 9H).

The sulfinamine (2.5 g, 8.6 mmol) was dissolved in 1,4-dioxane (60 mL) and HCl (8 mL, 4N in 1,4-dioxane) was added dropwise. After stirring for 1 hour at room temperature, the solvent was removed *in vacuo*, water (40 mL) and DCM (40 mL) were added and the organic layer discarded. The pH of the aqueous layer was adjusted to ~14 with NaOH pellets, extracted with DCM (2 x 50 mL) and the solvent removed to give the product amine as a clear oil (1.6 g, 100%).

^1H NMR (400 MHz, Chloroform-*d*) δ 7.35 (d, *J* = 8.7 Hz, 1H), 6.82 (d, *J* = 2.6 Hz, 1H), 6.75 (dd, *J* = 8.6, 2.6 Hz, 1H), 4.45 – 4.38 (m, 1H), 3.72 (s, 3H), 1.30 (d, *J* = 6.6 Hz, 3H).

(6-Fluoroquinoline-2-carbonyl)glycine

6-Fluoroquinoline-2-carboxylic acid (7 g, 37 mmol) and glycine methyl ester hydrochloride (5.1 g, 40 mmol) were dissolved in DCM (180 mL) and DIPEA (14.8 mL, 80.5 mmol) and cooled to 0 ˚C. Over 30 minutes PyBOP (21 g, 40 mmol) was added portionwise to the solution and stirring continued for 16 h allowing the reaction to warm to rt. The yellow solution was then concentrated *in vacuo* and the resulting oil diluted in ethyl acetate (300 mL). The organic solution was then washed with water (4 x 100 mL), dried over $MgSO_4$ and purified by FC (SiO_2). The resultant methyl ester was dissolved in a MeOH, THF, water mix (1:2:1; 120 mL) and lithium hydroxide added. The solution was stirred for 1h before concentrating in vacuo and acidifying with HCl (3M aqueous). The suspension was then extracted with ethyl acetate (3 x 150 mL) and the combined organics dried and concentrated to give product (7.6 g, 84%) as a white solid.

^1H NMR (400 MHz, Chloroform-*d*) δ 8.65 – 8.57 (m, 1H), 8.30 (dd, *J* = 8.5, 0.8 Hz, 1H), 8.25 (dd, *J* = 8.6, 0.8 Hz, 1H), 8.14 (ddt, *J* = 9.3, 5.5, 0.7 Hz, 1H), 7.54 (ddd, *J* = 9.2, 8.2, 2.8 Hz, 1H), 7.48 (dd, *J* = 8.7, 2.8 Hz, 1H), 4.24 (d, *J* = 5.5 Hz, 2H).

LCMS *m/z* 249.0 $(M+H)^+$

Benzyl (2*S*,4*R*)-1-((6-fluoroquinoline-2-carbonyl)glycyl)-4-hydroxypyrrolidine-2-carboxylate
Prepared according to general procedure B using (6-fluoroquinoline-2-carbonyl)glycine (900 mg, 3.6 mmol), benzyl (2*S*,4*R*)-4-hydroxypyrrolidine-2-carboxylate (1 g, 4.0 mmol), DIPEA (2.4 mL, 18 mmol), DCM (20 mL) and PyBOP (1.9 g, 4.0 mmol), purified by FC (SiO_2, 2-10% MeOH in DCM) to give the product (1.6 g, 98%) as a white solid.
^{1}H NMR (400 MHz, Chloroform-*d*) δ 8.99 – 8.85 (m, 1H), 8.31 – 8.20 (m, 2H), 8.21 – 8.12 (m, 1H), 7.54 (ddd, *J* = 9.3, 8.2, 2.8 Hz, 1H), 7.48 (dd, *J* = 8.7, 2.7 Hz, 1H), 7.43 – 7.26 (m, 5H), 5.29 – 5.13 (m, 2H), 4.76 (t, *J* = 8.0 Hz, 1H), 4.64-4.61 (m, 1H), 4.44 (d, *J* = 4.8 Hz, 0.4H), 4.39 (d, *J* = 4.8 Hz, 0.6H), 4.28 (d, *J* = 4.5 Hz, 0.6H), 4.24 (d, *J* = 4.5 Hz, 0.4H), 3.80 (d, *J* = 4.4 Hz, 0.4H), 3.78 (d, *J* = 4.3 Hz, 0.6H), 3.71 – 3.60 (m, 1H), 2.42-2.36 (m, 1H), 2.09 (ddd, *J* = 13.3, 8.0, 4.8 Hz, 2H).

(2*S*,4*R*)-1-((6-fluoroquinoline-2-carbonyl)glycyl)-4-hydroxypyrrolidine-2-carboxylic acid
To a solution of benzyl (2*S*,4*R*)-1-((6-fluoroquinoline-2-carbonyl)glycyl)-4-hydroxypyrrolidine-2-carboxylate (1.1g, 2.45 mmol) in MeOH (24 mL) was added palladium (10% activated on charcoal; 100 mg). The resultant mixture was hydrogenated for 16 h before filtering through celite (MeOH eluent) and concentrating *in vacuo*. Purification by FC (SiO_2; 2-20% MeOH in DCM + 1% acetic acid) gave the title compound (850 mg, 97%) as a white solid.
^{1}H NMR (400 MHz, Chloroform-*d*) δ 8.90 (q, *J* = 6.8, 5.8 Hz, 1H), 8.15 (d, *J* = 8.6 Hz, 1H), 8.08 (dd, *J* = 8.6, 0.8 Hz, 1H), 8.01 (dd, *J* = 9.3, 5.2 Hz, 1H), 7.46 – 7.33 (m, 2H), 4.56 – 4.44 (m, 1H), 4.46 – 4.37 (m, 1H), 4.30 – 4.18 (m, 1H), 4.16 – 4.05 (m, 1H), 3.69 – 3.58 (m, 1H), 3.51 – 3.41 (m, 1H), 2.25 – 2.15 (m, 1H), 2.06 – 1.94 (m, 1H), 1.36 – 1.17 (m, 1H).
LCMS *m/z* 362.2 $(M+H)^+$

***N*-(2-((2*S*,4*R*)-2-(((*S*)-1-(2-chloro-4-methoxyphenyl)ethyl)carbamoyl)-4-hydroxypyrrolidin-1-yl)-2-oxoethyl)-6-fluoroquinoline-2-carboxamide CAM833A**
(2*S*,4*R*)-1-((6-fluoroquinoline-2-carbonyl)glycyl)-4-hydroxypyrrolidine-2-carboxylic acid (3.6 g, 10.1 mmol) and (*S*)-1-(2-chloro-4-methoxyphenyl)ethan-1-amine (1.6 g, 8.61 mmol) were dissolved in DCM (50 mL), DIPEA (8.0 mL, 45.9 mmol) was added and the reaction mixture was cooled to 0 ˚C. HBTU (4.0 g. 10.6 mmol) was added portion-wise over 30 mins and the reaction mixture was allowed to warm to room temperature and stirred for 3 hours. The resulting precipitate was removed by filtration, and dissolved in DCM (200 mL) and washed with water (200 mL). Some precipitation occurred during the aqueous wash, which was collected by filtration, dissolved in ethyl acetate (150 mL) and washed with water (2 x 100 mL). The DCM organic layer was concentrated, and ethyl acetate (150 mL) was added and washed with water (2 x 100 mL). The organic layers were combined, dried with brine and magnesium sulfate and the solvent removed *in vacuo* to give the product as a white solid (2.8 g, 61%).
^{1}H NMR (500 MHz, DMSO-*d6*) δ 9.00-8.94 (m, 1H), 8.83 (d, J = 7.5 Hz, 0.3H), 8.58 (d, J = 8.5 Hz, 1H), 8.44 (d, J = 7.5 Hz, 0.7H), 8.25-8.18 (m, 2H), 7.93 (dd, J = 9.0, 2.5 Hz, 1H), 7.82 (m, 1H), 7.36 (d, J = 8.5 Hz, 0.3H), 7.29 (d, J = 8.5 Hz, 0.7H), 7.00 (d, J = 2.5 Hz, 0.3H), 6.95 (d, J = 2.5 Hz, 0.7H), 6.94 (dd, J = 2.5 Hz, 0.3H), 6.86 (dd, J = 8.5 Hz, 2.5 Hz, 0.7H), 5.19 (m, 1H), 5.08 (m, 0.7H), 4.60 (t, J = 7.5 Hz, 0.3H), 4.42 (t, J = 7.5 Hz, 0.7H), 4.35 (m, 0.7H), 4.27-4.12 (m, 2H), 3.87 (dd, 17.0, 5.5 Hz, 0.3H), 3.75 (s, 0.9H), 3.71 (s, 2.1H), 3.70-3.39 (m, 2H), 2.25 (m, 0.3H), 2.06 (m, 0.7H), 1.94 (m, 0.3H), 1.78 (m, 0.7H), 1.39 (d, J = 7.0 Hz, 0.9H), 1.30 (d, J = 7.0 Hz, 2.1H).
LCMS *m/z* 529.3 $(M+H)^+$
